# Epigenetics of Epileptogenesis-Evoked Upregulation of Matrix Metalloproteinase-9 in Hippocampus

**DOI:** 10.1371/journal.pone.0159745

**Published:** 2016-08-09

**Authors:** Katarzyna Zybura-Broda, Renata Amborska, Magdalena Ambrozek-Latecka, Joanna Wilemska, Agnieszka Bogusz, Joanna Bucko, Anna Konopka, Wieslawa Grajkowska, Marcin Roszkowski, Andrzej Marchel, Andrzej Rysz, Lukasz Koperski, Grzegorz M. Wilczynski, Leszek Kaczmarek, Marcin Rylski

**Affiliations:** 1 Department of Clinical Cytology, Centre of Postgraduate Medical Education, Warsaw, Poland; 2 Laboratory of Neurobiology, Nencki Institute of Experimental Biology PAS, Warsaw, Poland; 3 Laboratory of Molecular and Systemic Neuromorphology, Nencki Institute of Experimental Biology PAS, Warsaw, Poland; 4 Department of Pathology, The Children’s Memorial Health Institute, Warsaw, Poland; 5 Department of Neurosurgery, The Children’s Memorial Health Institute, Warsaw, Poland; 6 Department of Neurosurgery, Medical University of Warsaw, Warsaw, Poland; 7 Department of Pathology, Medical University of Warsaw, Warsaw, Poland; Augusta University, UNITED STATES

## Abstract

Enhanced levels of Matrix Metalloproteinase-9 (MMP-9) have been implicated in the pathogenesis of epilepsy in humans and rodents. Lack of Mmp-9 impoverishes, whereas excess of Mmp-9 facilitates epileptogenesis. Epigenetic mechanisms driving the epileptogenesis-related upregulation of MMP-9 expression are virtually unknown. The aim of this study was to reveal these mechanisms. We analyzed hippocampi extracted from adult and pediatric patients with temporal lobe epilepsy as well as from partially and fully pentylenetetrazole kindled rats. We used a unique approach to the analysis of the kindling model results (inclusion in the analysis of rats being during kindling, and not only a group of fully kindled animals), which allowed us to separate the molecular effects exerted by the epileptogenesis from those related to epilepsy and epileptic activity. Consequently, it allowed for a disclosure of molecular mechanisms underlying causes, and not consequences, of epilepsy. Our data show that the epileptogenesis-evoked upregulation of Mmp-9 expression is regulated by removal from Mmp-9 gene proximal promoter of the two, interweaved potent silencing mechanisms–DNA methylation and Polycomb Repressive Complex 2 (PRC2)-related repression. Demethylation depends on a gradual dissociation of the DNA methyltransferases, Dnmt3a and Dnmt3b, and on progressive association of the DNA demethylation promoting protein Gadd45β to Mmp-9 proximal gene promoter *in vivo*. The PRC2-related mechanism relies on dissociation of the repressive transcription factor YY1 and the dissipation of the PRC2-evoked trimethylation on Lys27 of the histone H3 from the proximal *Mmp-9* promoter chromatin *in vivo*. Moreover, we show that the DNA hydroxymethylation, a new epigenetic DNA modification, which is localized predominantly in the gene promoters and is particularly abundant in the brain, is not involved in a regulation of MMP-9 expression during the epileptogenesis in the rat hippocampus as well as in the hippocampi of pediatric and adult epileptic patients. Additionally, we have also found that despite of its transient nature, the histone modification H3S10ph is strongly and gradually accumulated during epileptogenesis in the cell nuclei and in the proximal Mmp-9 gene promoter in the hippocampus, which suggests that H3S10ph can be involved in DNA demethylation in mammals, and not only in *Neurospora*. The study identifies *MMP-9* as the first protein coding gene which expression is regulated by DNA methylation in human epilepsy. We present a detailed epigenetic model of the epileptogenesis-evoked upregulation of *MMP-9* expression in the hippocampus. To our knowledge, it is the most complex and most detailed mechanism of epigenetic regulation of gene expression ever revealed for a particular gene in epileptogenesis. Our results also suggest for the first time that dysregulation of DNA methylation found in epilepsy is a cause rather than a consequence of this condition.

## Introduction

One of the most prominent pathologic features of epilepsy is aberrant synaptic plasticity [1]. The synaptic plasticity–related protease Matrix Metalloproteinase-9 (MMP-9) [2–3] is an important stimulant for the development of epilepsy in humans and rodents [4–5]. It is upregulated in epilepsy; lack of Mmp-9 impoverishes, whereas excess of Mmp-9 facilitates epileptogenesis [4].

Mechanisms controlling the upregulation of Mmp-9 expression during epileptogenesis and in epilepsy are unknown. Here, we have investigated epigenetic regulation of Mmp-9 gene expression during epileptogenesis with a special emphasis on DNA methylation-dependent processes. Changes in DNA methylation are strongly involved in physiological and aberrant synaptic plasticity as well as in epilepsy development [6–7].

Herein, we report that the epileptogenesis-evoked upregulation of Mmp-9 expression in hippocampus is the consequence of a complex epigenetic mechanism involving strong and continuous demethylation of its proximal gene promoter, pronounced changes in transcriptionally repressive and activating histone modifications occurring in the chromatin of the promoter, as well as a regulatory action of the transcription factor YY1, acting in concert with the PRC2.

## Materials and Methods

### Human tissue

Human epileptic hippocampi (Table 1) included in this study were surgically removed from 13 patients who underwent surgical treatment for intractable epilepsy at the Children’s Memorial Health Institute, Warsaw, Poland, and the Department of Neurosurgery, Medical University of Warsaw, Poland. The control group consisted of 10 age- and localization-matched autopsy hippocampi obtained from patients who were free of the nervous system diseases. All these cases were reviewed by a neuropathologist. The fresh-frozen samples were derived from children (four focal cortical dysplasia (FCD) and three autopsy samples) and adult (nine FCD and seven autopsy samples) hippocampi. The human study protocol was in accordance with the Declaration of Helsinki and complied with the Polish national laws and the guidelines for the conduct of research that involves human subjects established by the Ethics Committees on Human Research at the Children’s Memorial Health Institute and Medical University of Warsaw, based on national laws. The human studies were approved by the Ethics Committee on Human Research at the Children’s Memorial Health Institute and the Ethics Committee on Human Research at the Medical University of Warsaw.

**Table 1:** Comparison of clinical data of epileptic patients and autopsy controls.

	Study group	Number of patients	Age (years)*		Pathological diagnosis
			Median	Range	
Pediatric patients	Epileptic	4	16	3.2–18.0	Hippocampal sclerosis/FCD
	Control	3	0.6	0.01–3	No pathological changes in the hippocampus
Adult patents	Epileptic	9	31	23–41	Hippocampal sclerosis/FCD
	Control	7	55	45–67	No pathological changes in the hippocampus

* Age at surgery for epileptic patients, and life span for control patients; FCD—focal cortical dysplasia

### Animals

Prior to the experiment, adult male Wistar rats (weighing 200–230 g) were habituated to handling for a week, and later they were injected intraperitoneally with saline three times every other day to reduce stress on the rat during the kindling phase. Food and water were available *ad libitum*. All procedures on rats were performed in accordance to the rules established by the IV Local Ethical Committee on Animal Research of the Centre of Medical Postgraduate Education, based on the national laws. The animal studies were approved by the IV Local Ethics Committee on Animal Research at the Centre of Medical Postgraduate Education. All efforts were made to minimize animal sufferings.

### Pentylenetetrazole (PTZ)-induced kindling

Rats received multiple intraperitoneal injections of PTZ (Sigma-Aldrich, St. Louis, MO, USA) in a subthreshold dose (30 mg/kg of rat body weight, PTZ dissolved in 0.9% sterile saline) every other day, consecutively up to 5 weeks. 30 mg/kg of PTZ is a nonseizure inducing dose, but after multiple administrations it finally led to a precipitation of seizures and to a gradual progression in their strength and duration. Behavioral seizures were scored according to a modified Racine’s scale [4]. Rats were observed up to 2 h after each injection. Animals were assembled into three groups: control (no PTZ injection, only saline injections instead), partially kindled (only weak seizures after repeated PTZ administrations), and fully kindled (strong seizures during the last three PTZ injections). To differentiate effects related to a single neuronal depolarization from effects related to epileptogenesis, we used additional control in our kindling experiment—rats injected with the single subthreshold PTZ dose. All animals were sacrificed 24 h after the last injection and both hippocampi were collected.

### Dizocilpine treatment and pentylenetetrazole (PTZ)-induced kindling

Rats were divided randomly into two groups and received every other day intraperitoneal injections of saline or dizocilpine (Abcam, Cambridge, UK; 0.1 mg/kg of rat body weight, dizocilpine hydrogen maleate dissolved in 0.9% sterile saline) 30 min before each PTZ injection, consecutively up to 5 weeks. Rats were observed up to 2 h after each PTZ injection. Behavioral seizures were scored as described earlier. All animals were sacrificed 24 h after the last injection and both hippocampi were collected.

### RNA extraction

RNA was extracted as we described previously [8].

### Reverse transcription quantitative PCR (RT-qPCR)

One microgram of RNA samples were subjected to RT reaction using TaqMan Reverse Transcription Kit (Applied Biosystem, Grand Island, NY, USA) according to enclosed procedure. Real-time PCR analysis was performed with 5× HOT FIREPol EvaGreen qPCR Mix Plus (Solis BioDyne, Tartu, Estonia) using the LightCycler480 System (Roche, Mannheim, Germany). Equal amounts of RNA were analyzed in triplicate, three times for each probe used. *C*_T_ values were chosen in the linear range of amplification, and the comparative *C*_T_ method was used to calculate differences in gene expression between samples [9–10].

### Nuclear protein lysates

Nuclear protein lysates were prepared as we described previously [8].

### Gelatin zymography

It was performed as we described previously [11]. As a control served activity of Mmp‐2 which is observed on zymography gels simultaneously to activity of Mmp‐9.

### DNA extraction

DNA was extracted using phenol:chloroform:isoamyl alcohol (25:24:1, Sigma-Aldrich) with additional incubation with RNase A at 37°C for 1 h.

### Chromatin immunoprecipitation (ChIP)

ChIP was performed as described previously [12].

### Methylated DNA immunoprecipitation (MeDIP) with subsequent qPCR

MeDIP protocol was adapted from Thu and colleagues [13]. One microgram of human or rat DNA from each sample was sheared to an average size of 500–1000 bp fragments by a sonication (Cole-Parmer, Vernon Hills, IL, USA, ultrasonic Processor, confirmed by a gel agarose analysis), and subsequently divided into immunoprecipitated (IP) and input samples (IN). IP samples were incubated with an anti-5-methylcytosine antibody (Calbiochem, Darmstadt, Germany).

### Hydroxymethylated DNA immunoprecipitation (hMeDIP) with subsequent qPCR

hMeDIP protocol was analogous with MeDIP although anti-5-hmC antibody (Active Motif, Carlsbad, CA, USA) and Dyneabeads M-280 anti-rabbit IgG (Invitrogen, Carlsbad, CA, USA) were used.

### Bisulfite conversion (BC)

Following isolation and purification of DNA from rat hippocampal tissue, bisulfite modification was performed using EpiTect Bisulfite Kit (Qiagen, Germantown, MD, USA) according to manufacturer’s procedure. The amount of 1.5 μg of DNA was converted in each reaction. Then, the DNA was applied to EpiTect spin columns (Qiagen) for purification. The eluted DNA was checked for the efficiency of conversion using PCR amplifying Mmp‐9 gene proximal promoter fragment, which has C (not placed within CpG sequence) in the primer binding sites. Purified samples of converted DNA were used for the methylation‐specific PCR (MSP) and bisulfite sequencing (BSP).

### Subcloning of bisulfite-converted DNA and sequencing (BSP)

Bisulfite-treated samples of DNA were PCR amplified. Primers were hand-designed to fit the proximal promoter fragment of rat Mmp-9 gene. Primers were complementary to sequences of bisulfite modified DNA, and did not contain CpG sites in their sequence. The PCR products were purified using QIAquick PCR purification Kit (Qiagen) and subcloned using pGEM-T Easy Vector System II (Promega, Madison, WI, USA). Plasmid DNA was isolated using PureYield Plasmid Miniprep System (Promega). Up to 20 clones for each amplicon in each study group were sequenced by Genomed Company. CloneManager software (Scientific & Educational Software, Morrisville, NC, USA) was used for all molecular cloning‐related *in silico* analyses. BiQ Analyzer [14] was used to align sequences and perform quality control tests for sequencing, conversion errors, and clonality.

### Methylation-specific PCR (MSP)

Sodium bisulfite–modified DNA was PCR amplified using two pairs of primers for each studied CpG site (one pair was specific for the methylated DNA, and the other for the unmethylated DNA) with EpiTect MSP Kit (Qiagen). β-tubulin-4 levels were used for normalization. Amplified products were analyzed by agarose gel electrophoresis. Agarose gel resolved products were analyzed using Gene Snap (Syngene) and Bio Imaging System (Syngene).

### Western blotting (WB)

Western blotting was performed as we described previously [8].

### Antibodies for WB and ChIP

See S1 Table.

### RT-qPCR and ChIP primers and PCR conditions

See S2 Table.

### MeDIP, BC, BS, and MSP primers, and PCR conditions

See S3 Table.

### Statistical analyses

Statistical significance was determined using a nonparametric, 2-tailed Mann–Whitney *U* test for data with non-normal distribution. The Kolmogorov–Smirnov test and Shapiro–Wilk test were used to validate the assumption of normality. Parametric student’s *t* test (to compare two groups) or 1-way ANOVA (for three or more groups) was used for data with normal distribution. Results are shown as mean ± SEM. Differences were considered significant at *p* < 0.05. Statistical analysis was conducted with Statistica (StatSoft).

## Results

### In the hippocampus of epileptic patients, MMP-9 promoter demethylation is associated with MMP-9 gene activation

**Fig 1:**
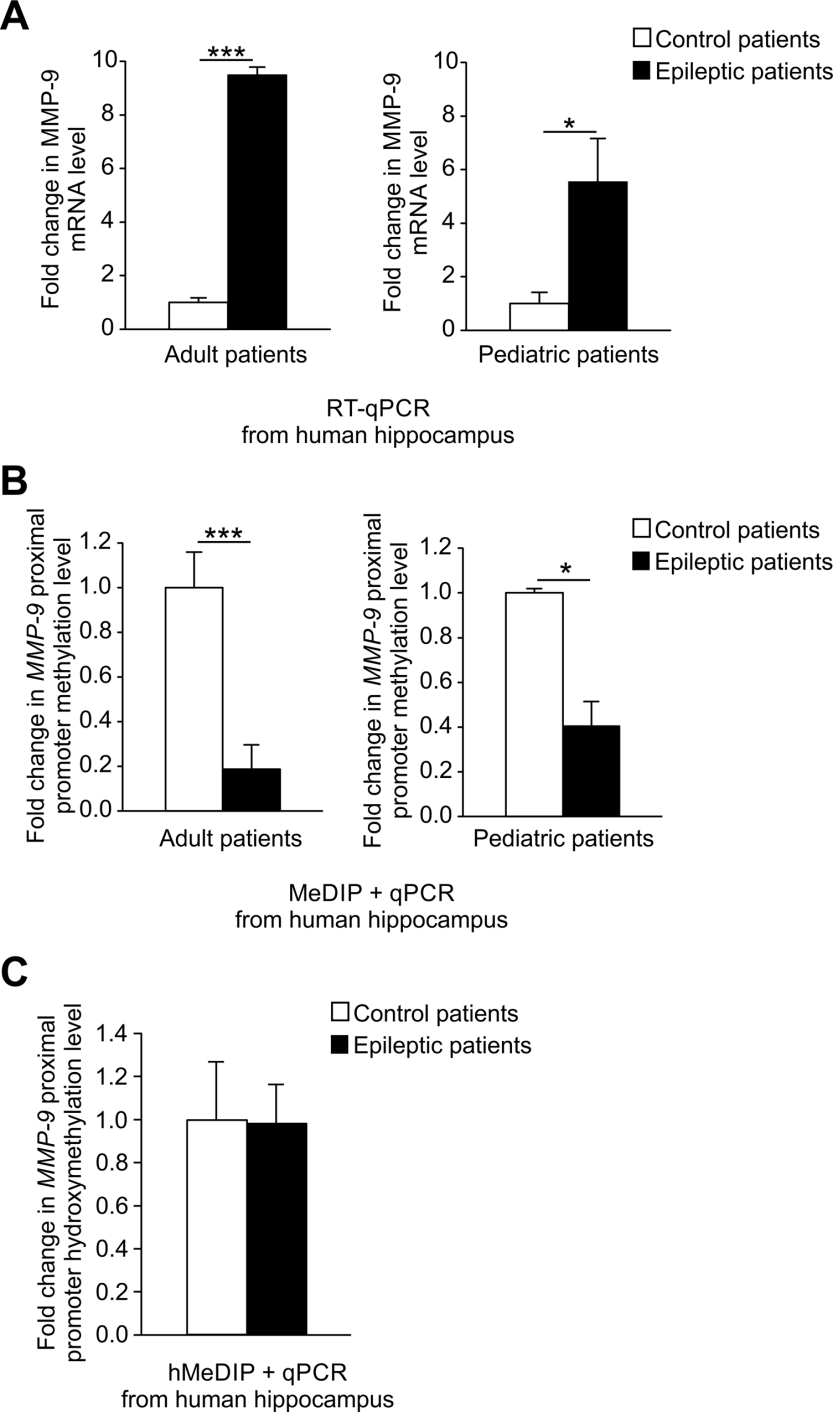
MMP-9 mRNA upregulation is accompanied by robust *MMP-9* promoter demethylation in hippocampi of epileptic patients. **(A) *MMP-9 mRNA expression is substantially increased in the hippocampi of adult and pediatric epileptic patients***. For each RT-qPCR analysis, equal amounts of RNA samples isolated from control, epileptic adult and pediatric patients’ hippocampi were used. Data is presented as fold change in mRNA expression. Values are means ± SEM (*, *p* < 0.05; ***, *p* < 0.001; *n* = 6 for adults, *n* = 3–4 for pediatric patients). (**B) *MMP-9 proximal promoter methylation is strongly diminished in vivo in the hippocampi of epileptic patients***. *MMP-9* proximal promoter methylation level was evaluated by qPCR using DNA samples obtained by the immunoprecipitation of methylated DNA (MeDIP) coming from hippocampi of control as well as adult and pediatric epileptic patients. Data is presented as fold change in the methylation status. Values are means ± SEM (*, *p* < 0.05; ***, *p* < 0.001; *n* = 6 for adults, *n* = 3–4 for pediatric patients). **(C) *MMP-9 proximal promoter is hydroxymethylated at similar level in vivo in epileptic and control patients***. Level of *MMP-9* proximal promoter hydroxymethylation was evaluated using qPCR with DNA samples obtained from immunoprecipitation of hydroxymethylated DNA (hMeDIP) coming from hippocampi of control and epileptic patients. Data is presented as fold change in hydroxymethylation status. Values are means ± SEM (*n* = 6).

To determine whether MMP-9 is abnormally expressed in patients with the mesial temporal sclerosis, we analyzed MMP-9 mRNA levels using reverse transcription quantitative PCR (RT-qPCR) in the samples of hippocampi surgically dissected from adult and pediatric epileptic patients as well as from autopsy controls, who died from disorders unrelated to the nervous system (Table 1). Neuropathologist’s examination revealed that all hippocampi were cortical dysplasia–free, but displayed sclerotic features. We observed a strong increase in MMP-9 mRNA expression in both adult and pediatric epileptic patients compared to autopsy controls (Fig 1A). The more prominent increase in MMP-9 mRNA level was detected in adult epileptic patients compared to pediatric ones (Fig 1A).

DNA methylation of gene promoters is a potent transcriptional regulatory mechanism inversely correlated to gene expression. To evaluate the possibility that this phenomenon is involved in the control of MMP-9 mRNA upregulation in human epilepsy, we investigated by methylated DNA immunoprecipitation (MeDIP) whether the proximal *MMP-9* promoter was demethylated in epileptic patients. We observed significant decrease in *MMP-9* methylation level *in vivo* in the hippocampi of both adult and pediatric epileptic patients compared to autopsy controls (Fig 1B). Moreover, DNA demethylation was stronger in adult compared to pediatric patients, what convincingly correlated with MMP-9 mRNA expression profiles (compare Fig 1A to Fig 1B).

DNA hydroxymethylation is a recently discovered epigenetic DNA modification implicated in transcriptional regulation, and especially in DNA-demethylation-dependent activation of gene expression [15–16]. To probe whether hydroxymethylation of *MMP-9* proximal promoter is potentially capable of regulating MMP-9 gene activation in epileptic patients, we applied the hydroxymethylated DNA immunoprecipitation (hMeDIP). The results showed similar and significant enrichment of *MMP-9* proximal promoter fragments *in vivo* in epileptic and control patient samples compared to their respective isotype controls (Fig 1C). These findings imply no involvement of the DNA hydroxymethylation in the activation of MMP-9 expression in human epilepsy.

To check the reliability of autopsy samples used as control materials for the experiments shown in Fig 1, we have analyzed whether tissue preservation process, analogical to the one applied to a preservation of autopsy samples used in our study, could alter the MMP-9 mRNA expression level and the degree of the *MMP-9* proximal promoter methylation. To this end, RNA and DNA were isolated immediately from the rat hippocampi, as well as at 72 hours after the animal sacrifice. We observed no difference between the two study groups in the Mmp-9 mRNA expression and the *Mmp-9* proximal promoter methylation status (S1 Fig, panel A and B). This result confirms credibility of autopsy controls used in our experiments.

### Epileptogenesis-related gradual MMP-9 gene promoter demethylation progressively upregulates MMP-9 expression

To examine the mechanism of DNA demethylation-dependent upregulation of MMP-9 expression in epilepsy, we proceeded with the analyses of animal epileptogenesis model, known as pentylenetetrazole-induced kindling (PTZ-kindling). Previously, Mizoguchi *et al*. demonstrated that MMP-9 protein expression and its gelatynolytic activity are increased in the hippocampi of PTZ-fully kindled mice [17].

**Fig 2:**
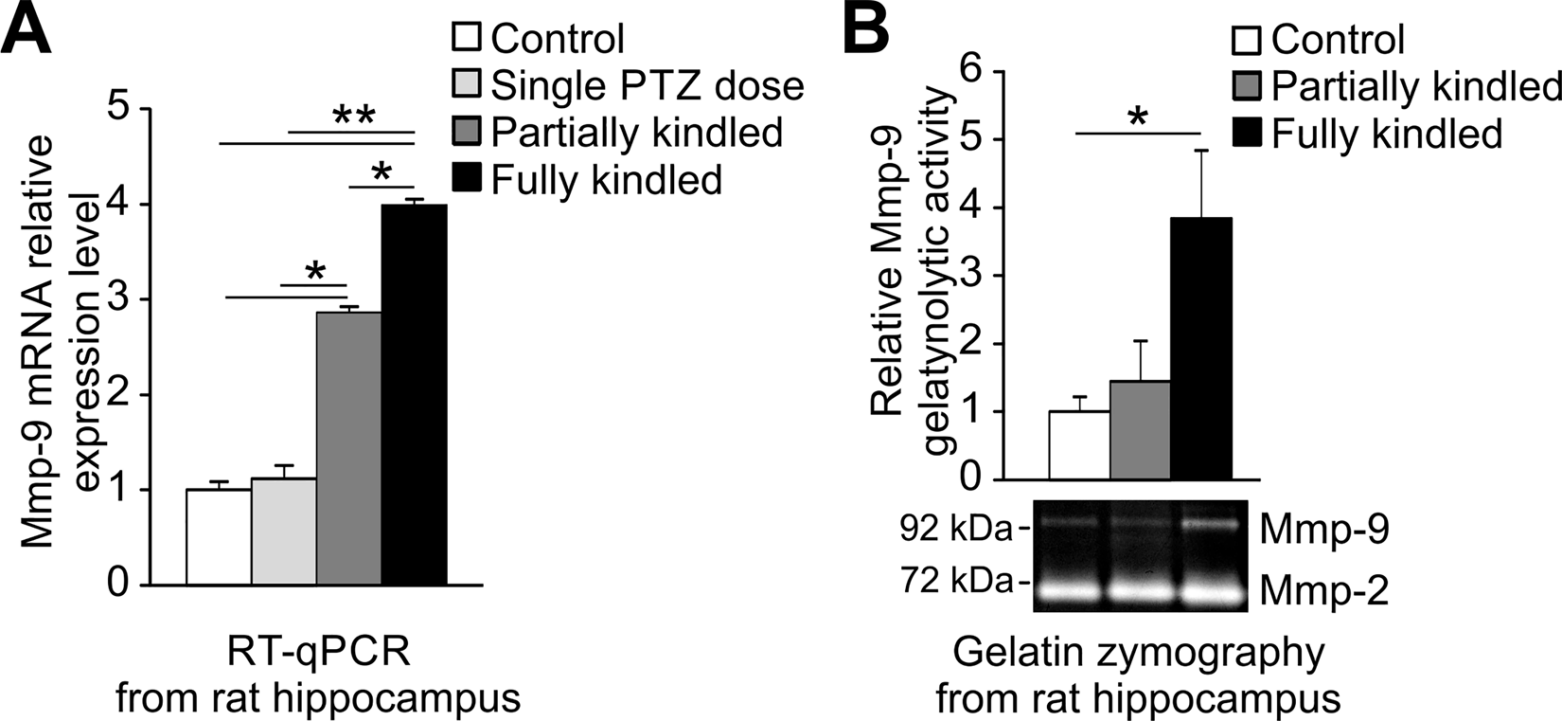
Mmp-9 mRNA expression and activity are increased progressively during epileptogenesis in the rat hippocampus. 30 mg/kg of PTZ was administrated intraperitoneally at least 10 times to partially and fully kindled study group. Single PTZ dose study group received only one PTZ administration at 30 mg/kg dose. Rats were sacrificed 24 h after the final dose. (**A) *Mmp-9 mRNA accumulates progressively during epileptogenesis in the hippocampus***. For each analysis equal amounts of RNA samples isolated from naive (control) and PTZ-treated (single PTZ dose, partially kindled, fully kindled) rat hippocampi were used. Data is presented as fold change in mRNA expression. Values are means ± SEM (*, *p* < 0.05; **, *p* < 0.01; *n* = 4). (**B) *Mmp-9*, *but not Mmp-2*, *gelatynolytic activity augments strongly in the hippocampi of PTZ-kindled rats***. All of the study groups showed unchanged hippocampal MMP-2 activity, whereas MMP-9 activity was substantially modified. For gelatine zymography analysis equal amounts of protein samples isolated from naive (control) and PTZ-treated (single PTZ dose, partially and fully kindled) rat hippocampi were used. Graph data is presented as fold change in gelatynolytic activity. Values are means ± SEM (*, *p* < 0.05; *n* = 4). Representative cropped gel image of Mmp-9 and Mmp-2 gelatynolytic activity is showed.

Here, we examined, by RT-qPCR, Mmp-9 mRNA changes occurring in the hippocampi of partially and fully kindled rats during PTZ-induced epileptogenesis (Fig 2A).

We found that Mmp-9 mRNA expression was gradually upregulated (Fig 2A). Moreover, one PTZ subthreshold dose administration had no influence on Mmp-9 mRNA expression, which confirmed that our observations were related to multiple PTZ administrations, and thus related to epileptogenesis (Fig 2A). Next, we investigated how changes in Mmp-9 mRNA are accompanied by alternations in the Mmp-9 protein activity. We performed gelatin zymography studies. We observed that the Mmp-9 mRNA upregulation during epileptogenesis is accompanied by a significant increase in Mmp-9 enzymatic activity (Fig 2B).

**Fig 3:**
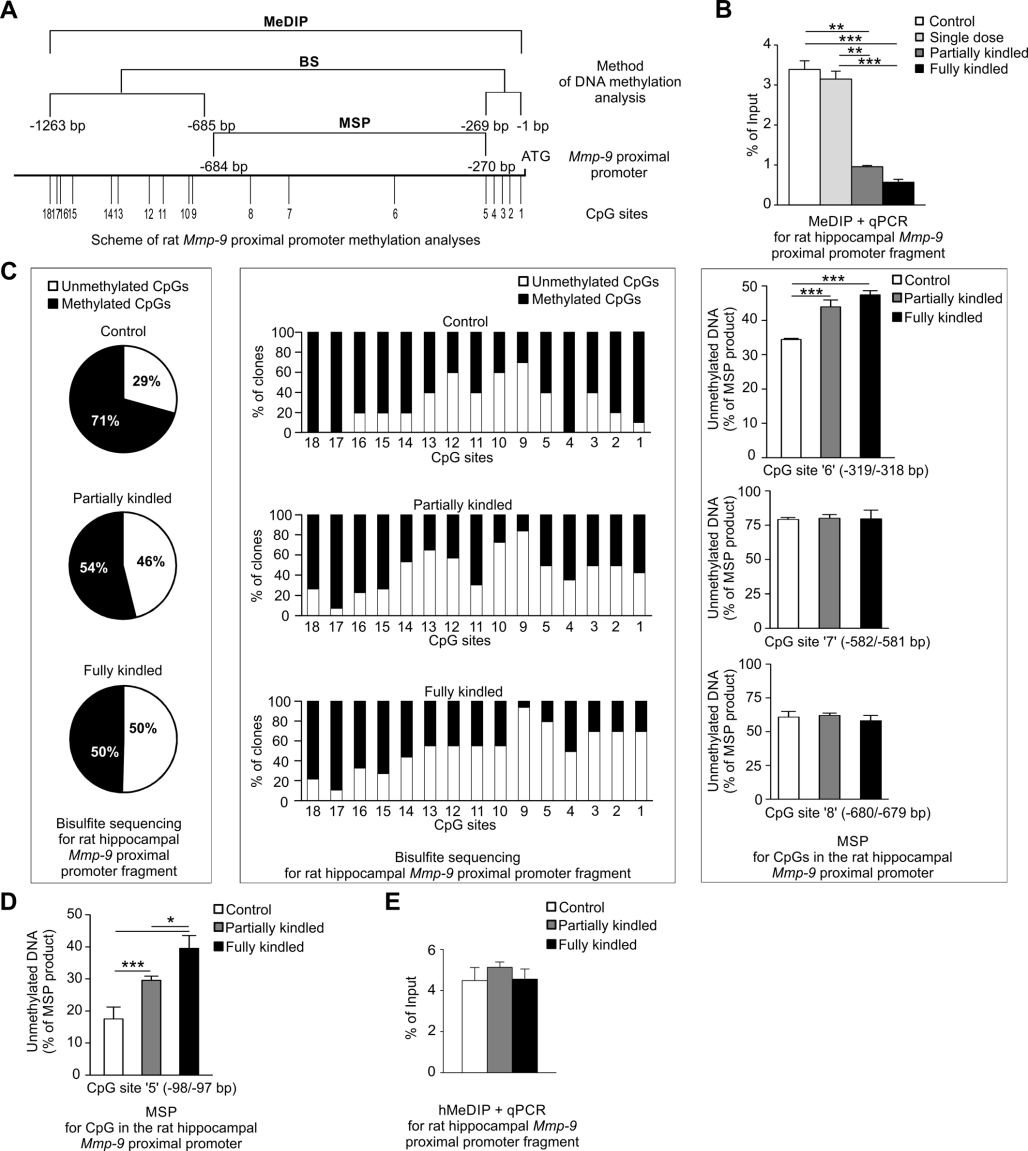
*Mmp-9* proximal promoter is progressively demethylated during PTZ-induced epileptogenesis in the rat hippocampus. **(A) *Schematic presentation of our experimental approach to a detailed evaluation of the rat Mmp-9 proximal promoter methylation*.** *In vivo* methylation status of ~1000 bp fragment of the *Mmp-9* proximal promoter was analyzed using the methylated DNA immunoprecipitation method (MeDIP). Methylation of CpG sites localized in the -1263/-685 and the -269/-1 bp fragments of the proximal *Mmp-9* promoter (sites 1–5 and 9–18 in the scheme) were analyzed *in vitro* by bisulfite sequencing. The methylation status of CpG sites situated in the -684/-270 bp fragment of the proximal *Mmp-9* promoter (sites 6–8 at the scheme) were evaluated *in vitro* using methylation-specific PCR (MSP). Additionally, to provide a control for the consistency of bisulfite sequencing results and data obtained by MSP, the -98/-97bp CpG site of the *Mmp-9* promoter was evaluated using both these procedures (compare middle panel in Fig 3C and 3D). (**B) *Mmp-9 proximal promoter is demethylated during epileptogenesis in vivo*.** Methylation level was revealed by MeDIP and presented as % of input. (**C) *Mmp-9 proximal promoter is progressively demethylated in most of its CpG sites during epileptogenesis in vitro*.** Methylation of CpG sites localized in -1263/-685 and -269/-1 bp fragments of *Mmp-9* promoter was evaluated using bisulfite sequencing. Graph data is presented as % of the total number of methylated and unmethylated CpG sites found in all examined clones in these regions (left panel) or as % of the total number of methylated and unmethylated CpG sites detected in the particular CpG site in the promoter fragments in all analyzed clones (central panel). Right panel presents results of DNA methylation at CpG sites located in the -684/-270 bp fragment of *Mmp-9* promoter using MSP. Data is presented as % of unmethylated DNA. (**D) *The -98/-97bp CpG site of Mmp-9 promoter is gradually demethylated during epileptogenesis in vitro*.** Methylation level was analyzed by MSP (presented as % of unmethylated DNA). (**E) *DNA hydroxymethylation of rat hippocampal Mmp-9 proximal promoter remains unchanged during epileptogenesis in vivo*.** Hydroxymethylation was assessed by hMeDIP and data is presented as % of input. In Fig 3B, 3C (right panel), 3D and 3E values are means ±SEM and *n* = 4. In Fig 3B and 3C (right panel) **, *p*<0.01; ***, *p*<0.001. In Fig 3D *, *p*<0.05; ***, *p*<0.001.

Later, we evaluated DNA methylation and DNA hydroxymethylation status of the *Mmp-9* proximal promoter in rat hippocampus during PTZ-induced epileptogenesis *in vivo* and *in vitro*. These DNA methylation analyses were multiprocedural and very detailed, the scheme of which is shown and described in Fig 3A. First, we checked the level of DNA methylation present in ~1000 bp fragment of the *Mmp-9* proximal promoter *in vivo* using MeDIP (Fig 3B). We observed strong DNA demethylation during epileptogenesis in the gene promoter *in vivo* (Fig 3B).

There are 18 CpG sites localized in the -1263/-1 bp *Mmp-9* promoter fragment, which can be potentially and differentially methylated. To perform the proximal *Mmp-9* promoter fragment DNA methylation studies in detail, we performed bisulfite sequencing (BSP) and/or methylation-specific PCR (MSP) (Fig 3C and 3D).

BSP was used to investigate the methylation changes occurring in 15 CpG sites located in the -1263/-685 and the -269/-1 bp *Mmp-9* rat promoter fragments. We found that a percentage of a total number of the demethylated CpG sites substantially increased during epileptogenesis from 29% in control animals, through 46% in partially kindled, to 50% in fully kindled rats (Fig 3C, left panel). Moreover, further detailed and more thorough evaluation of the BSP results showed that 14 out of 15 CpG sites contained in the -1263/-685 and -269/-1 bp *Mmp-9* rat promoter fragments were demethylated during epileptogenesis (Fig 3C, middle panel). The following were the CpG sites: -11/-10, -38/-37, -57/-56, -78/-77, -98/-97, -824/-823, -832/-831, -898/-897, -1011/-1010, -1028/-1027, -1125/-1124, -1156/-1155, -1160/-1159, and -1173/-1174 bp. Among these demethylated CpG sites, progressive demethylation occurred during epileptogenesis at the sites localized in the most proximal promoter, (-11/-10, -38/-37, -57/-56, -78/-77, -98/-97 bp CpG sites), and also in the -824/-823, -1125/-1124, -1156/-1155, -1160/-1159 bp CpG sites. Demethylation of the -1011/-1010 bp and the -1028/-1027 bp CpG sites was the highest in partially kindled rats and partially reduced in epileptic rats; however, it was still far higher than in control animals. Demethylation of the -898/-897 bp CpG site was the highest in epileptic rats, but during epileptogenesis it was reduced. The -934/-933 bp CpG site was the only one in which methylation was maintained in a stable level during epileptogenesis.

To evaluate *in vitro* methylation status of the -684/-270 bp fragment of the *Mmp-9* promoter, we applied MSP. We found that the -319/-318 bp CpG site was gradually demethylated, while methylation level of the -582/-581 and -680/-679 bp CpG sites remained stable during epileptogenesis (Fig 3C, right panel, and Fig 3D). For confirmation of mutual accordance of BSP results with MSP data, we additionally analyzed the -98/-97 bp CpG site methylation status using MSP. This site was chosen because it demonstrated clear and gradual DNA methylation changes during epileptogenesis in BSP evaluation. MSP, like BSP, also demonstrated gradual demethylation of the -98/-97 bp CpG site during epileptogenesis, suggesting reciprocal congruity of MSP and BSP results (compare Fig 3C, middle panel to Fig 3D). Collectively, BSP and MSP results indicated that progressive epileptogenesis-related DNA demethylation of the hippocampal *Mmp-9* proximal promoter principally concentrated in its two regions: the most proximal -319/-10 bp fragment of the promoter and the -1160/-1124 bp region.

DNA hypomethylation occurs during normal aging [18]. Thus, we tested whether the age of rats before and after the kindling experiment (12- and 17-week old rats) could have impact on our results. We observed no difference in the Mmp-9 mRNA expression and the *Mmp-9* proximal promoter methylation between the two study groups (S2 Fig, panel A and B).

Then, we checked whether changes in *Mmp-9* proximal promoter methylation are limited, similarly as in humans (Fig 1C), to changes in 5-mC or also include modulation of 5-hmC content. We revealed by hMeDIP that DNA hydroxymethylation of the rat hippocampal *Mmp-9* proximal promoter remained unchanged during epileptogenesis *in vivo* (Fig 3E).

Later, we have hypothesized that the epileptogenesis-evoked augmentation of Mmp-9 expression in the hippocampus is dependent on demethylation of its proximal gene promoter. Consequently, blockage of epilepsy development should lead to an inhibition of epileptogenesis-dependent demethylation of *Mmp-9* promoter, and consequently to prevention of Mmp-9 mRNA upregulation.

Regulation of Mmp-9 expression and development of epilepsy are both NMDA receptor–dependent [19–22]. We used dizocilpine, the NMDA receptor antagonist displaying anticonvulsant activity, to block the development of PTZ-evoked epilepsy to check whether epileptogenesis inhibition had the ability to stop PTZ-kindling-dependent induction of Mmp-9 promoter demethylation and Mmp-9 expression.

**Fig 4:**
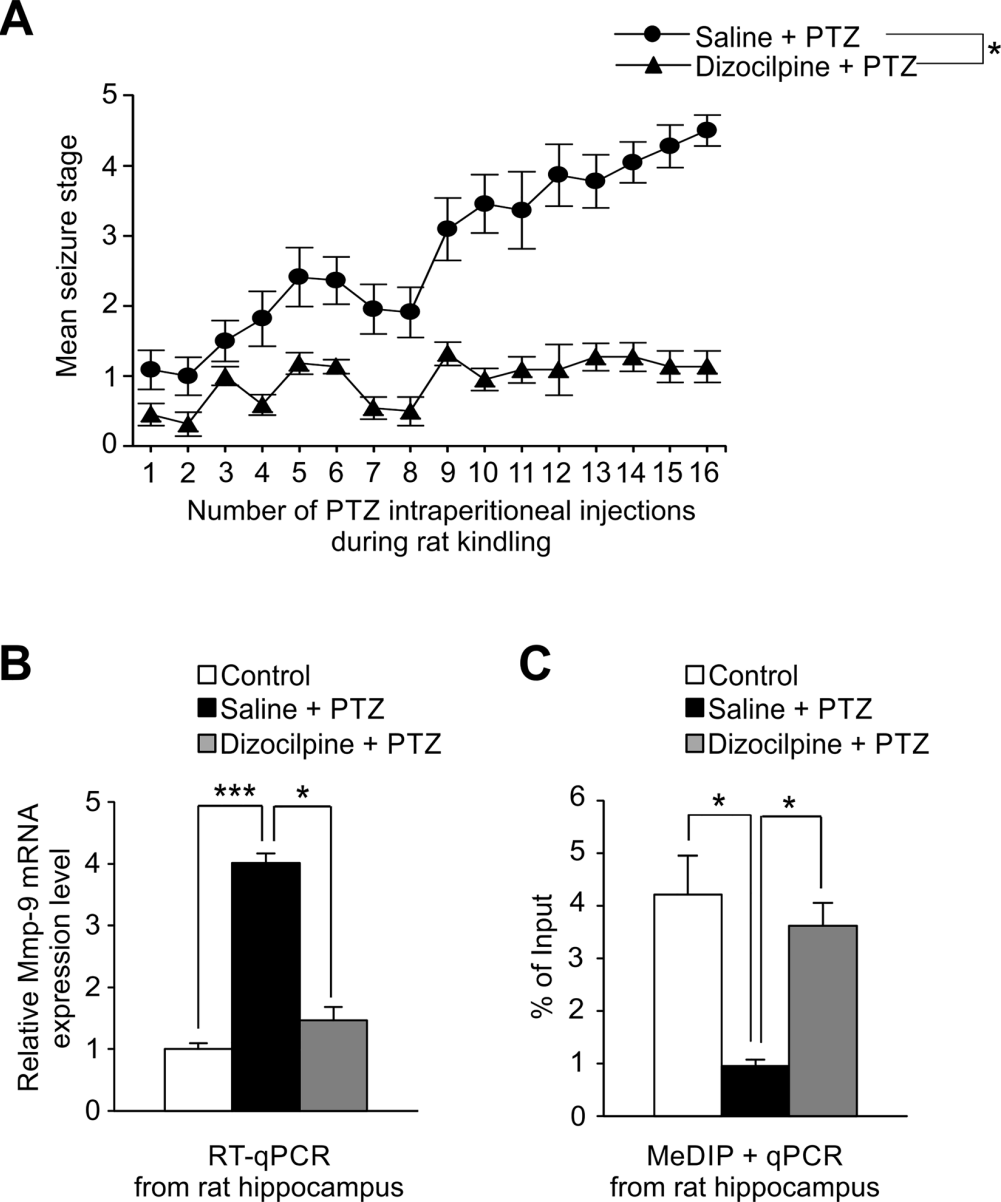
Mmp-9 upregulation during epilepsy development is strictly dependent on epileptogenesis-evoked demethylation of its gene promoter. To block epilepsy development, we used dizocilpine (the NMDA receptor antagonist displaying anticonvulsant activity). 0.1 mg/kg of dizocilpine or saline was intraperitoneally injected to rats 30 min before each PTZ dose administration (30 mg/kg). (**A) *Dizocilpine treatment effectively suppresses the development of PTZ-evoked epilepsy in rats***. Animals were observed up to 2 h after each PTZ injection and seizures were scored according to a modified Racine’s scale. Values are means ± SEM (*, *p*<0.05; *n* = 11). (**B) *PTZ kindling-evoked upregulation of the Mmp-9 mRNA expression is fully inhibited by dizocilpine administration in the rat hippocampus*.** Dizocilpine administration suppresses the PTZ kindling–evoked augmentation in the hippocampal Mmp-9 mRNA expression, whereas repeated PTZ treatment without dizocilpine injections leads to significant upregulation of Mmp-9 mRNA level. For RT-qPCR analysis equal amounts of RNA isolated from naive (control), PTZ-treated (saline + PTZ), and dizocilpine-treated (dizocilpine + PTZ) rat hippocampi were used. Data is presented as fold change in mRNA expression. Values are means ± SEM (*, *p*<0.05; ***, *p*<0.001; *n* = 8). **(C) *Dizocilpine treatment completely inhibits the PTZ kindling-dependent Mmp-9 proximal promoter demethylation in the rat hippocampus***. *Mmp-9* proximal promoter methylation level was evaluated using qPCR in DNA samples obtained by immunoprecipitation of methylated DNA from naive (control), PTZ-treated (saline+PTZ), and dizocilpine-treated (dizocilpine+PTZ) rat hippocampi. Data is presented as a percent of input. Values are means ± SEM (*, *p*<0.05; *n* = 5).

It has been demonstrated previously that dizocilpine inhibits PTZ-kindled sizures as well as an upregulation of the hippocampal Mmp-9 protein activity in mice [17]. Similarly, our behavioral analyses showed that dizocilpine treatment effectively suppressed the development of PTZ-evoked epilepsy in the rat hippocampus (Fig 4A). Moreover, dizocilpine-dependent inhibition of epileptogenesis also fully prevented PTZ kindling–evoked demethylation of *Mmp-9* proximal promoter (Fig 4B), suggesting that this demethylation is strictly related to epilepsy development. Consequently, dizocilpine-evoked suppression of epileptogenesis completely blocked the PTZ kindling–evoked upregulation of Mmp-9 mRNA expression (Fig 4C).

### Epileptogenesis-evoked *Mmp-9* promoter demethylation depends on coordinated action of Dnmt3a, Dnmt3b and Gadd45β

Next, we asked what is a molecular mechanism of the epileptogenesis-related DNA demethylation of the proximal *Mmp-9* promoter in the hippocampus. The degree of DNA methylation found in gene promoters depends mainly on a coordinated action of DNA methyltransferases as well as DNA demethylases acting principally in the proximal regions of the promoters.

**Fig 5:**
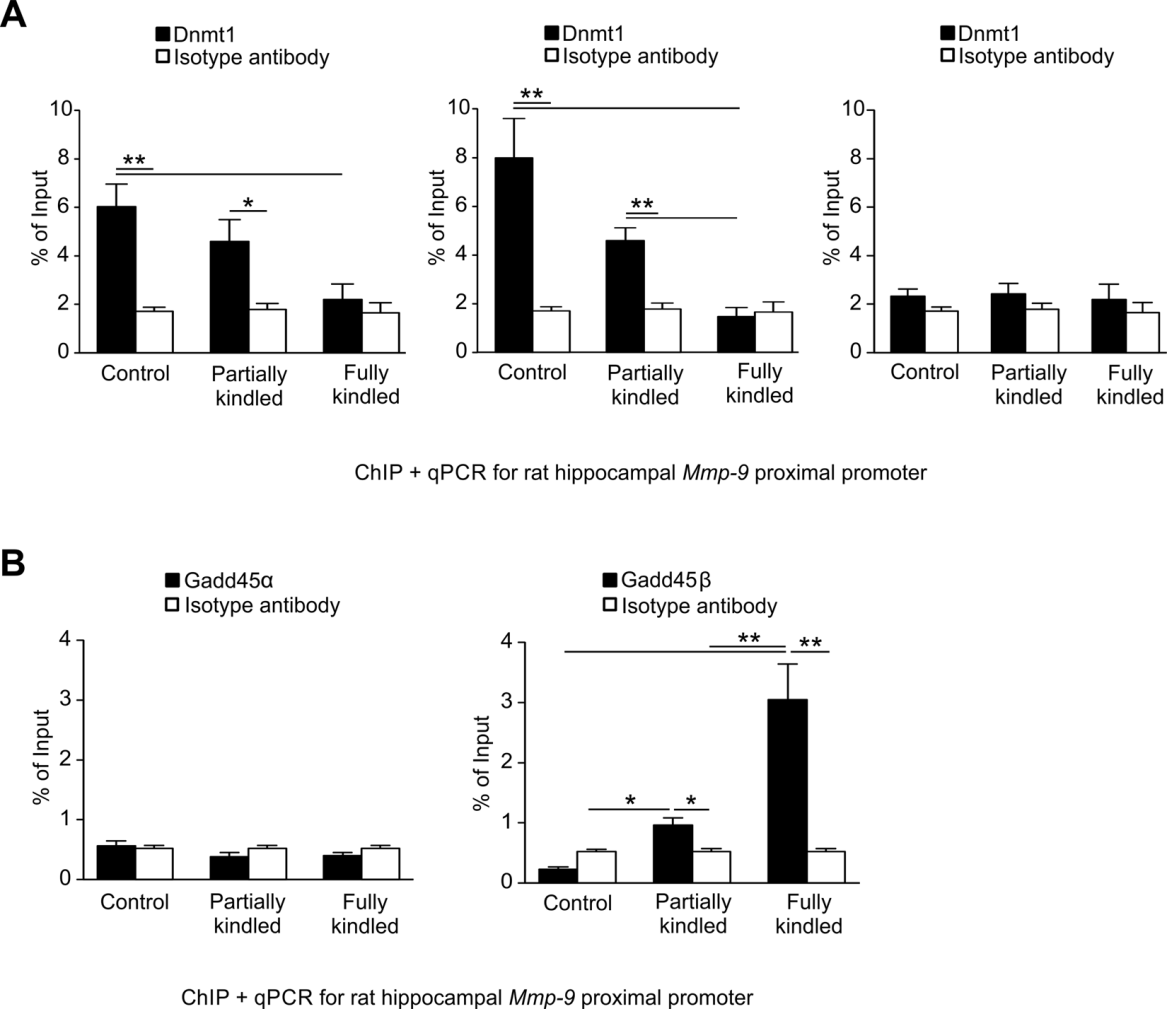
Coordinated action of Dnmt3a, Dnmt3b and Gadd45β regulates the epileptogenesis-evoked Mmp-9 promoter demethylation in hippocampus. **(A) *Dnmt3a and Dnmt3b*, *but not Dnmt1*, *bind to the proximal Mmp-9 promoter in the unstimulated rat hippocampus*, *and are gradually dissociating from it during the hippocampal epileptogenesis in vivo*.** DNA samples were obtained by chromatin immunoprecipitation with anti-Dnmt3a (left graph), anti-Dnmt3b (central graph), or anti-Dnmt1 (right graph) antibodies from the unstimulated (control), and the PTZ-kindled (partially kindled, fully kindled) rat hippocampi. *Mmp-9* proximal promoter content was evaluated by qPCR. Control ChIP reaction was performed using isotype antibody. Values are means ± SEM (*, *p* < 0.05; **, *p* < 0.01; *n* = 4). (**B) *The regulator of neuronal activity–dependent DNA demethylation Gadd45β*, *but not its functional and structural homologue Gadd45α*, *increasingly binds to the Mmp-9 proximal promoter during epileptogenesis in the rat hippocampus in vivo*.** DNA samples were obtained by chromatin immunoprecipitation with anti-Gadd45α (left graph) or anti-Gadd45β (right graph) antibodies from naive (control) as well as from the PTZ-treated (partially kindled, fully kindled) rat hippocampi. *Mmp-9* proximal promoter content was evaluated by qPCR. Control ChIP reaction was performed using isotype antibody. Values are means ± SEM (*, *p* < 0.05; **, *p* < 0.01; *n* = 4).

We looked for the expression profiles of DNA methyltransferases during epileptogenesis. We found that all three mammalian methyltransferases Dnmt1, Dnmt3a, and Dnmt3b were expressed in the hippocampus (S3 Fig). Moreover, we observed that their expression was stable and it did not change during epileptogenesis (S3 Fig). Then, we detected by chromatin immunoprecipitation (ChIP) a progressive dissociation of DNA methyltransferases Dnmt3a and Dnmt3b from the *Mmp-9* proximal promoter during hippocampal epileptogenesis *in vivo* (Fig 5A). Additionally, the data obtained revealed that Dnmt1 was not present in the chromatin of the *Mmp-9* proximal promoter, not participating in a regulation of *Mmp-9* promoter demethylation during epileptogenesis (Fig 5A).

Now, we argued whether DNA demethylation of the *Mmp-9* proximal promoter is just a passive mechanism related only to the dissociation of DNA methylation promoting proteins, such as Dnmt3a and Dnmt3b, or maybe it is a more complex process in which demethylating agents are actively involved too. DNA demethylases acting in neurons are largely unknown. The only one group of proteins which has been showed to demethylate DNA in neurons are TET enzymes [15] which oxidize 5-mC into 5-hmC [23]. However, their involvement in the regulation of the *Mmp-9* promoter demethylation is improbable, because of lack of changes in DNA hydroxymethylation localized in the *MMP-9* proximal promoter in the hippocampi of epileptic humans and rats during epileptogenesis (Figs 1C and 3E). Thus, we focused on Growth arrest and DNA-damage-inducible (Gadd45) protein family. Gadd45 proteins do not exhibit DNA demethylation activity *per se*, but Gadd45α and Gadd45β are DNA-binding components of multisubunit protein complexes displaying DNA demethylation activity, and interestingly Gadd45β has been showed to promote DNA demethylation in hippocampal neurons in response to neuronal activity [24].

We found that Gadd45α and Gadd45β are expressed at stable level during epileptogenesis in the hippocampus (S4 Fig). We also detected by ChIP that Gadd45α is not localized to the chromatin of the *Mmp-9* proximal promoter *in vivo* in all studied groups (Fig 5B). Similarly, there was no Gadd45β present in the chromatin of *Mmp-9* proximal promoter in unstimulated control hippocampi (Fig 5B). Intriguingly, however, we detected gradually increasing Gadd45β association into the chromatin of the *Mmp-9* proximal promoter in partially and fully PTZ-kindled rats (Fig 5B).

### YY1- and PRC2-related repression controls epileptogenesis-induced MMP-9 upregulation in the hippocampus

In the next experiment, we have asked whether epigenetic molecular mechanisms other than DNA methylation and DNA hydroxymethylation could be involved in a regulation of Mmp-9 expression during epilepsy development in the hippocampus. Previous studies showed that the transcription factor YY1 was a potent regulator of neuronal Mmp-9 expression during enhanced depolarization [8]. Interestingly, YY1 recruits epigenetically active PRCs to regulatory elements of DNA leading to an introduction of PcG-specific histone modifications and ultimately to a transcriptional repression [25].

**Fig 6:**
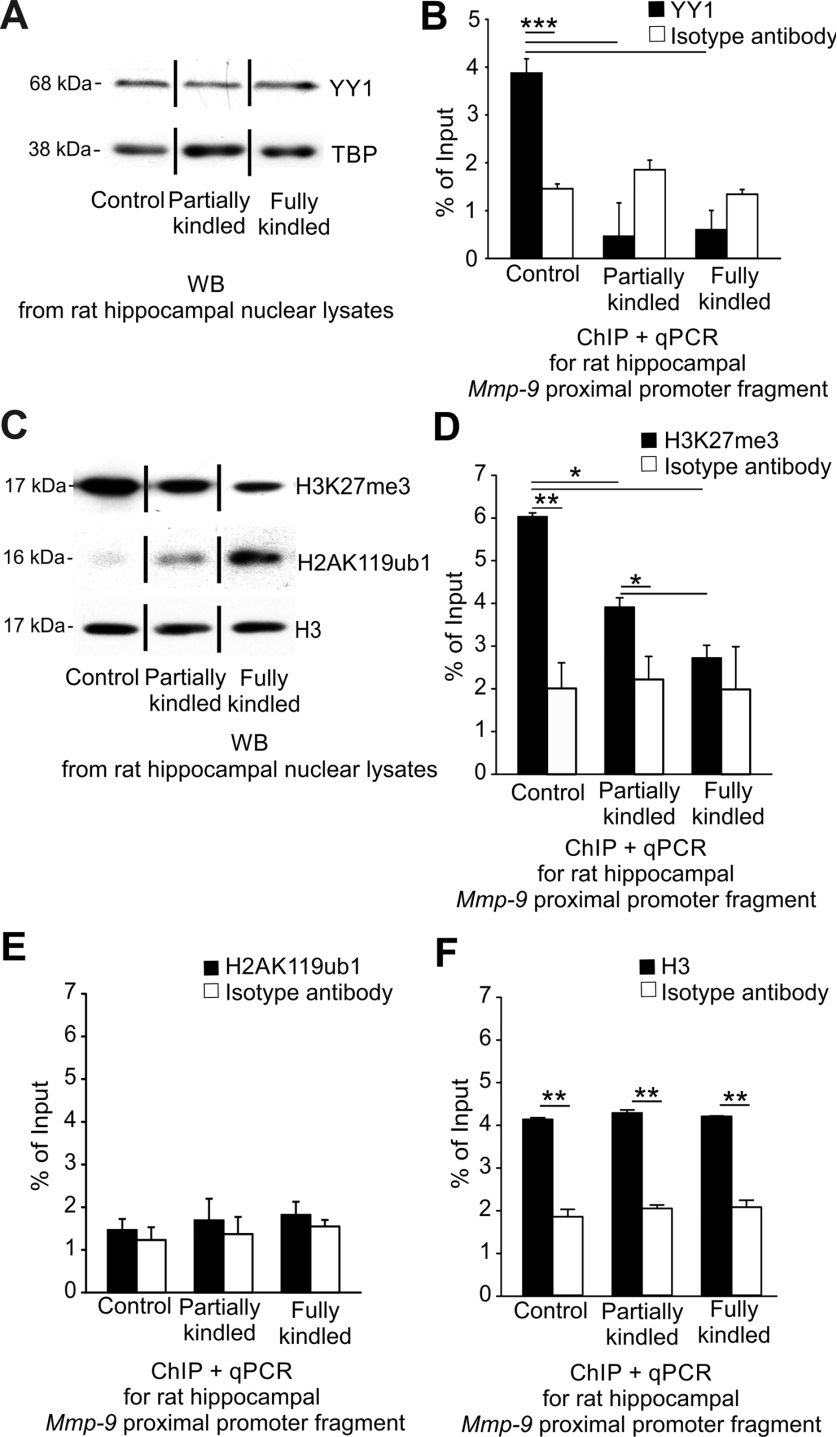
YY1- and PRC2-related repression controls epileptogenesis-induced MMP-9 upregulation in the hippocampus. **(A) *YY1 is expressed on a constant level during epileptogenesis in the rat hippocampus*.** Equal amounts (20 μg) of nuclear cell lysates obtained from the unstimulated (control) as well as PTZ-kindled (partially kindled, fully kindled) rat hippocampi were analyzed by Western blot with anti-YY1 antibody. As a loading control, TATA-binding protein (TBP) was used. Representative cropped Western blot analyses are shown. **(B) *YY1 binds to the proximal Mmp-9 promoter in the unstimulated rat hippocampus*, *and dissociates from it early during hippocampal epileptogenesis in vivo*.** DNA samples were obtained by chromatin immunoprecipitation with anti-YY1 antibody from the unstimulated (control), and the PTZ-kindled (partially kindled, fully kindled) rat hippocampi. The *Mmp-9* proximal promoter content was evaluated by qPCR. Control ChIP reaction was performed using isotype antibody. Values are means ± SEM (***, *p* < 0.001; *n* = 3). **(C) *The expression of histone H3 trimethylated on lysine 27 (H3K27me3) is decreased and histone H2A monoubiquitylated on lysine 119 (H2AK119ub1) is increased during PTZ-induced kindling*.** Equal amounts (20 μg) of the nuclear cell lysates were analyzed by Western blot. As a loading control, histone H3 was used. (**D) *During epileptogenesis in the rat hippocampus*, *PRC2-related transcriptionally repressive histone modification H3K27me3 is progressively decreased in chromatin of the proximal Mmp-9 promoter in vivo*.** DNA samples were chromatin immunoprecipitated using anti-H3K27me3 antibody and analyzed by qPCR for *Mmp-9* proximal promoter content. Values are means ±SEM (*, *p* < 0.05; **, *p* < 0.01; *n* = 4). (**E) *In the rat hippocampus*, *the PRC1-related transcriptionally repressive histone modification H2AK119ub1 is not present in the chromatin of the proximal Mmp-9 promoter in vivo in the unstimulated rats as well as in rats developing epilepsy*.** DNA samples were chromatin immunoprecipitated with anti-H2AK119ub1 antibody and analyzed by qPCR for *Mmp-9* proximal promoter content. Values are means ± SEM (*n* = 4). **(F) *Histone H3 is strongly and stably enriched in the chromatin of the proximal MMP-9 promoter in vivo during epileptogenesis in the rat hippocampus*.** DNA samples were chromatin immunoprecipitated with anti-histone H3 antibody and analyzed by qPCR for *Mmp-9* proximal promoter content. Values are means ± SEM (**, *p* < 0.01; *n* = 4).

We looked for YY1 involvement in the epileptogenesis-evoked transcriptional upregulation of Mmp-9 expression. We found that YY1 is stably expressed during the hippocampal PTZ-evoked epileptogenesis (Fig 6A). Subsequently, we detected strong epileptogenesis-induced dissociation of the repressive transcription factor YY1 from the *Mmp-9* proximal promoter chromatin occurring at early stages of epilepsy development by ChIP (Fig 6B).

Then, we have investigated whether YY1-binding is accompanied by the presence of Polycomb-related histone modifying enzymatic activities in the hippocampal chromatin of *Mmp-9* proximal promoter *in vivo*. Polycomb proteins repress gene expression, acting in two multisubunit protein complexes, namely PRC1 exhibiting activity for monoubiquitylation of histone H2A on lysine 119 (H2AK119ub1) and PRC2 catalyzing trimethylation of histone H3 on lysine 27 (H3K27me3) [26].

First, we found by Western blot that the hippocampal H3K27me3 expression was progressively downregulated and the hippocampal H2AK119ub1 expression was gradually increased during epileptogenesis in the hippocampus (Fig 6C). As a loading control for the experiments, Western blot for histone H3 was used (Fig 6C). Then consequently, we analyzed the occupancy of the hippocampal chromatin of the *Mmp-9* proximal promoter *in vivo* by H3K27me3 using ChIP. We found that there was a strong expression of H3K27me3 in the hippocampal chromatin of the *Mmp-9* proximal promoter of control rats, and a significant reduction of the expression in the partially kindled rats, whereas the fully kindled animals exhibited no H3K27me3 expression in the genome site (Fig 6D). Moreover, we studied the H2AK119ub1 accumulation in the hippocampal chromatin of the *Mmp-9* proximal promoter *in vivo* by ChIP, and we revealed that H2AK119ub1 was not expressed in the chromatin of the *Mmp-9* proximal promoter in any of the study groups (Fig 6E). As a control for the experiments presented in Fig 6D and 6E to show that our ChIP technique is properly working, we chromatin immunoprecipitated histone H3, which is constitutively expressed in a cell chromatin (Fig 6F).

In addition, we also checked by ChIP whether H3K9me2 and H3K9me3, the other histone modifications that are abundant in neurons and their progenitors exert transcriptionally repressive action, can be involved in a regulation of MMP-9 expression during the development of epilepsy *in vivo* [27–28]. We found that both H3K9me2 and H3K9me3 were not involved in a regulation of the Mmp-9 expression during epileptogenesis (S5 Fig).

### Upregulation of Mmp-9 expression is accompanied by H3S10ph accumulation in its gene promoter chromatin *in vivo*

We previously showed that Mmp-9 upregulation during enhanced neuronal depolarization is strictly coupled to the histone H3 and H4 deacetylation occurring in the chromatin of its proximal gene promoter [8]. Therefore, we have also checked if there was a similar situation during the epileptogenesis-evoked activation of Mmp-9 transcription.

First, we observed by Western blot that the hippocampal hyperacetylation of the histone H3 was diminished in partially and fully kindled rats as compared to the unstimulated ones, whereas we also found that the hippocampal hyperacetylation of the histone H4 was stable during the development of epilepsy (S6 Fig, panel A). Then surprisingly, we found by ChIP that there was no hyperacetylation of H3 or H4 in the *Mmp-9* proximal promoter chromatin during epileptogenesis *in vivo* in the rat hippocampus (S6 Fig, panel B and C).

**Fig 7:**
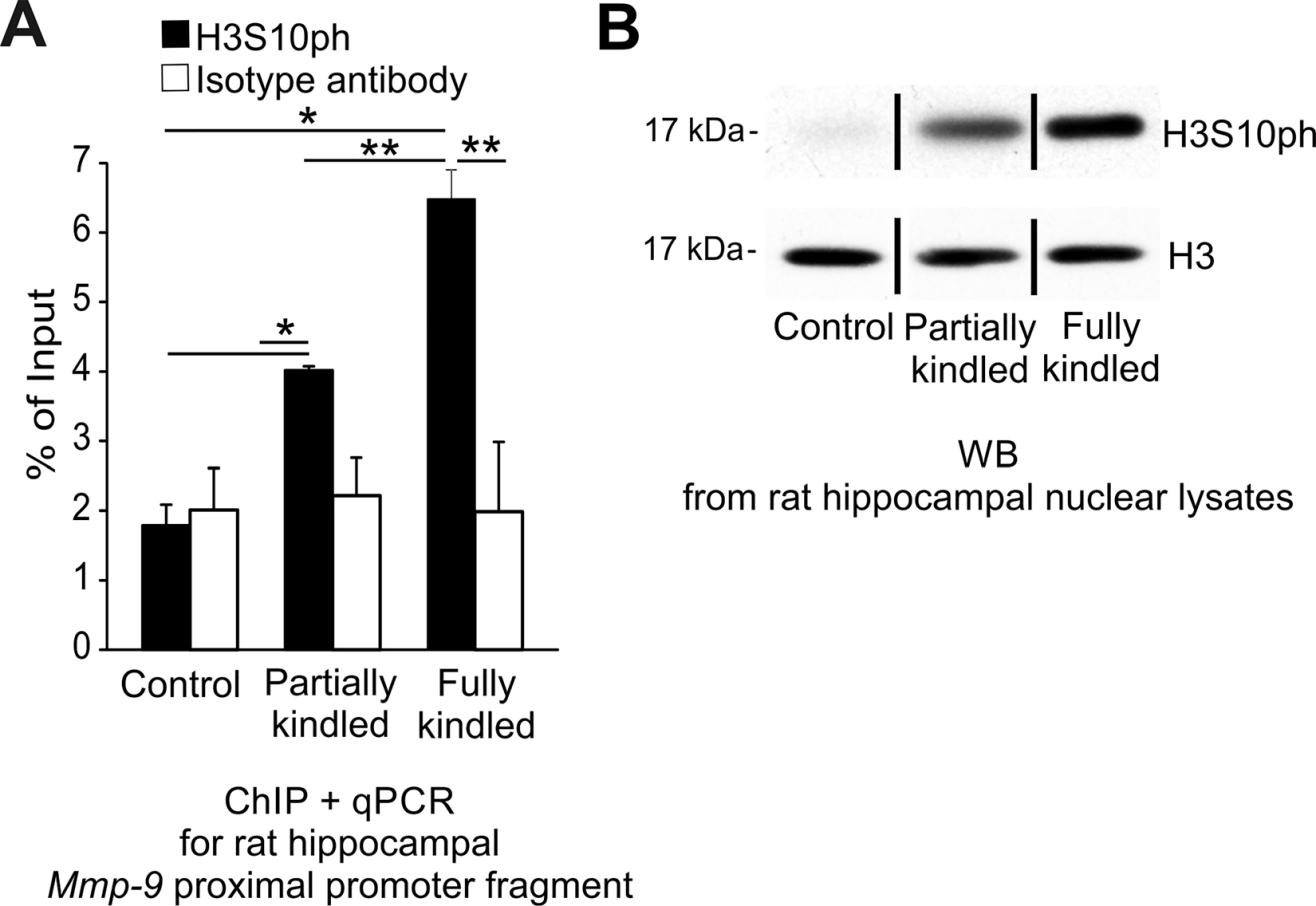
H3S10ph, transcriptionally activating histone modification, is strongly induced during epileptogenesis in the hippocampal chromatin. **(A) *The phosphorylation of the histone H3 on serine 10 (H3S10ph) gradually and strongly increases in chromatin of the Mmp-9 proximal promoter during epileptogenesis in the rat hippocampus in vivo*.** DNA was isolated from the hippocampal samples obtained by chromatin immunoprecipitation with anti-H3S10ph antibody from the unstimulated (control), as well as from the partially kindled and fully kindled rats. *Mmp-9* proximal promoter content was evaluated by qPCR. Control ChIP reaction was performed using isotype antibody. Values are means ± SEM (*, *p* < 0.05; **, *p* < 0.01; *n* = 4). (**B) *During epileptogenesis in the rat hippocampus*, *the histone H3 is strongly phosphorylated on serine 10*.** Equal amounts (20 μg) of nuclear cell lysates obtained from the unstimulated (control) as well as the partially kindled and fully kindled rat hippocampi were analyzed by Western blot with anti-H3S10ph antibody. As a loading control, histone H3 was used. Representative Western blot analyses are shown.

Antecedently, it has been shown that the acetylation on lysine 9 of the histone H3 (H3K9ac) [29], the dimethylation on lysine 4 of histone H3 (H3K4me2) [30], and the phosphorylation on the serine 10 of histone H3 (H3S10ph) are important for an upregulation of gene transcription in the brain [31–32]. Consequently, we asked whether H3K9ac, H3K4me2, or H3S10ph can be involved in the upregulation of the Mmp-9 mRNA during epileptogenesis in the hippocampus. We immunoprecipitated chromatin with anti-H3K9ac, anti-H3K4me2, and anti-H3S10ph antibody, but we did not detect the presence of either H3K9ac or H3K4me2 in hippocampal *Mmp-9* proximal promoter chromatin during the development of seizures (S6 Fig, panel D and E). Interesting, however, we observed the very strong progressive accumulation of H3S10ph in *Mmp-9* proximal promoter chromatin *in vivo* during epileptogenesis (Fig 7A). Similarly, we also noticed a substantial, general upregulation of the H3S10ph expression by Western blot during the development of epilepsy in the hippocampus (Fig 7B). Collectively, these results suggest that there exists robust, epileptogenesis-evoked phosphorylation on serine 10 of histone H3 in chromatin of the hippocampal cell nuclei, and particularly in the *Mmp-9* proximal promoter chromatin.

## Discussion

The deregulation of Mmp-9 enzymatic activity is an important molecular pathologic event facilitating the development of epilepsy [4]. Furthermore, it is strictly related to the upregulation of the Mmp-9 mRNA expression. Surprisingly, despite strong Mmp-9 involvement in animal epileptogenesis, there is no data about mechanisms regulating the epileptogenesis-evoked upregulation of Mmp-9 mRNA expression. In the present study, we investigated phenomena that might control the MMP-9 gene expression. We found that in the hippocampi of the adult and pediatric epileptic patients there was a strong demethylation of *MMP-9* proximal promoter *in vivo*, which led to a robust increase in MMP-9 mRNA expression. Additionally, we are the first who evaluated 5-hydroxymethylcytosine involvement in the epigenetic control of gene expression during epileptogenesis and in human temporal lobe epilepsy. Our results revealed that DNA hydroxymethylation, which is the other epigenetic mechanism capable of controlling brain gene expression, is not involved in the regulation of MMP-9 hippocampal expression in during rat epileptogenesis and in human temporal lobe epilepsy.

Later, searching for more precise mechanisms of MMP-9 expression regulation, we moved into a well-established model of epileptogenesis—the PTZ-kindling. Using this experimental design and applying multiple experimental approaches *in vivo* and *in vitro*, we observed the gradual demethylation of the proximal *Mmp-9* promoter during epileptogenesis in the hippocampus. Simultaneously, we detected the progressive epileptogenesis-evoked upregulation of Mmp-9 mRNA expression in the rat hippocampus, which was accompanied by the strong augmentation of hippocampal Mmp-9 enzymatic activity in the epileptic group of animals. Correspondingly, like in epileptic humans, there was no involvement in this process of the DNA hydroxymethylation-related mechanisms.

Moreover, we looked at the epileptogenesis-related changes of the methylation status in each CpG site located in the -1263/-1 *Mmp-9* proximal promoter fragment. We found that out of 18 CpG sites contained in the fragment, 15 were demethylated during epilepsy development, methylation of 3 CpG sites were unchanged, and there were no CpG sites, which would be progressively methylated during epileptogenesis in the hippocampus. Interestingly, the strongest, progressive, epileptogenesis-related demethylation was concentrated especially in two regions of the -1263/-1 *Mmp-9* proximal promoter fragment, namely in the CpG sites localized in the most proximal promoter (the -98/-10 bp promoter fragment) and in the -1160/-1124 fragment.

Later, we blocked epileptogenesis with NMDA receptor antagonist, and we showed that once there was no development of epilepsy, there was no *Mmp-9* proximal promoter demethylation, and accordingly there was no upregulation of Mmp-9 expression, which suggested a strict dependence of the Mmp-9 mRNA elevation on the demethylation of its gene promoter. Next, we demonstrated that the demethylation of the *Mmp-9* proximal promoter during epileptogenesis in the hippocampus *in vivo* is a consequence of two mechanisms. Firstly, the progressive reduction in the degree of the *Mmp-9* proximal promoter methylation due to the consecutive dissociation of *de novo* DNA methyltransferases Dnmt3a and Dnmt3b from the gene promoter chromatin. Secondly, the continuous augmentation of the active DNA demethylation of genome fragment as a result of gradual accumulation of the regulator of neuronal activity–dependent DNA demethylation Gadd45β in the Mmp-9 proximal promoter chromatin.

**Fig 8:**
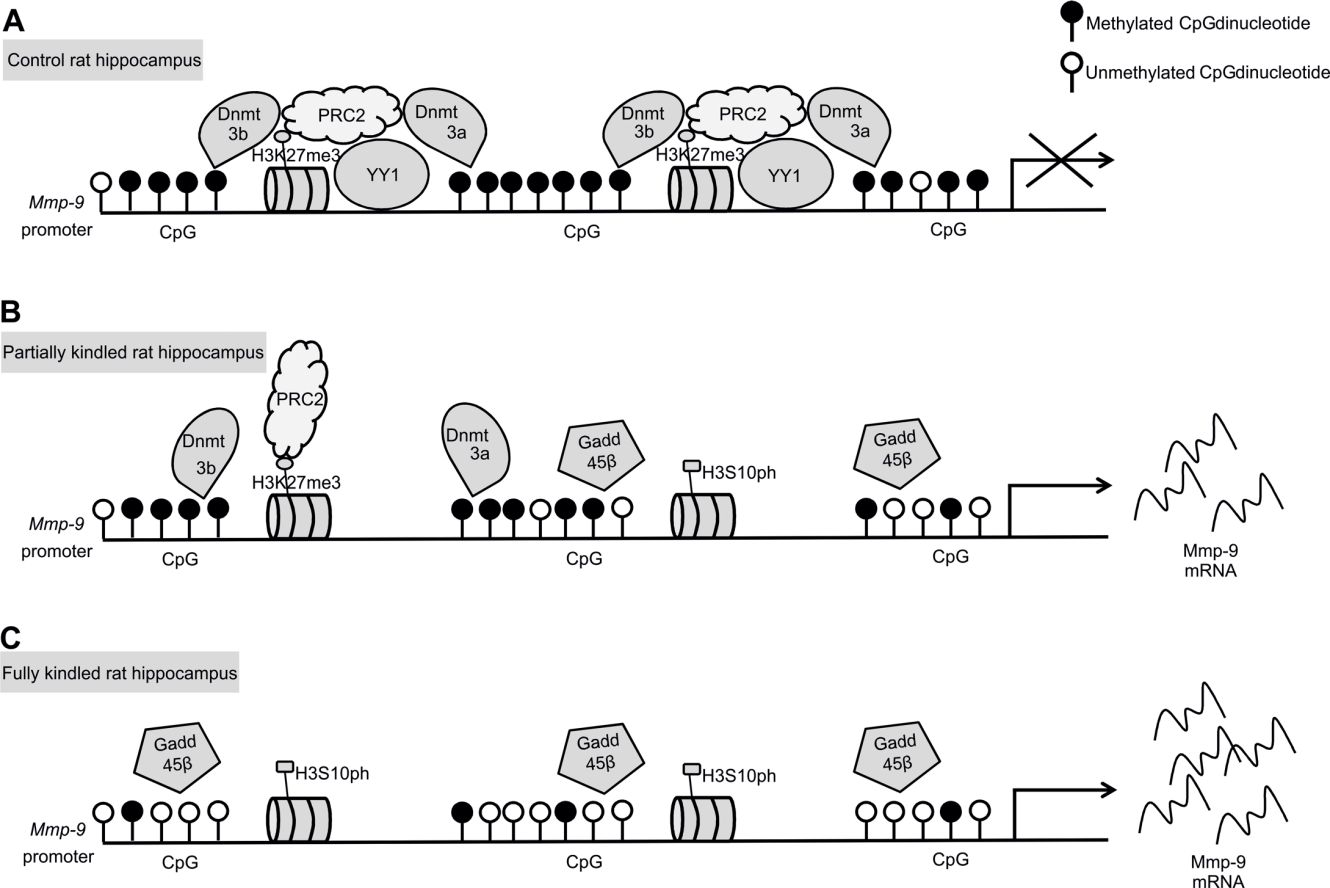
Model of the epileptogenesis-evoked upregulation of Mmp-9 expression in the hippocampus. **(A)** In the control rat hippocampus, Mmp-9 proximal promoter is bound by YY1, which nucleates the DNA methyltransferases, Dnmt3a and Dnmt3b, as well as PRC2, leading to the strong promoter DNA methylation and simultaneous trimethylation on lysine 27 of histone H3 in the surrounding chromatin. (**B)** In partially kindled rat hippocampus, YY1 dissociates out of the gene promoter, leading to a partial removal from the chromatin region other silencing proteins Dnmt3a, Dnmt3b, and PRC2. Concomitantly, DNA-demethylation-related protein Gadd45β and the activating histone mark H3S10ph start to accumulate in the chromatin. Consequently, it induces partial demethylation of the Mmp-9 gene promoter and leads to a reduction in its chromatin of the repressive chromatin mark H3K27me3. These complex molecular events lead to a moderate stimulation of the Mmp-9 gene expression. **(C)** In the fully kindled rat hippocampus, Mmp-9 expression is highly upregulated as a consequence of a much more profound proximal promoter demethylation (due to a complete dissociation from its chromatin of Dnmt3a and Dnmt3b as well as a strongly increased accumulation of Gadd45β), disappearance of the PRC2-related repression with a complete removal of H3K27me3, and a significant increase in the abundance of H3S10ph in the chromatin region.

Finally, we demonstrated that the repressive transcription factor YY1 dissociates from Mmp-9 proximal promoter chromatin during epileptogenesis in hippocampus *in vivo* leading to the dissipation of PRC2 activity from the chromatin region, what was reflected by a gradual reduction of histone modification H3K27me3 in the chromatin region. Moreover, we have also demonstrated no changes in H2AK119ub1 what excludes PRC1 involvement in the epileptogenesis-related regulation of Mmp-9 expression. Interestingly, the epileptogenesis-evoked upregulation of hippocampal Mmp-9 expression *in vivo* was also accompanied by the gradual accumulation of transcriptionally activating histone modification H3S10ph in the chromatin of its proximal gene promoter. Collectively, our data indicates that epileptogenesis-evoked upregulation of Mmp-9 mRNA expression in hippocampus is regulated by a complex mechanism related to interweaved action of at least the Dnmt3a, Dnmt3b, and Gadd45β-related demethylation of the *Mmp-9* proximal promoter as well as, dependent on the dissociation of transcription factor YY1, the dissipation of the PRC2-related histone modifying activity from the chromatin of the gene promoter and also the accompanying intense phosphorylation on serine 10 of the histone H3 in promoter chromatin. Therefore, we propose the model of gradual activation of Mmp-9 expression during hippocampal epileptogenesis presented graphically in Fig 8.

Our data shows that in the upregulation of Mmp-9 expression are involved the two potent repressor mechanisms–DNA methylation and PRC2-related repression, which are removed out of its proximal promoter. Intriguingly, it has been shown that PRC2 may direct DNA methylation to the specific genome sites due to the recruitment of DNA methyltransferases [33–34]. Thus, it is possible that in the unstimulated hippocampus one repressive complex exists composed of *de novo* Dnmts, PRC2, as well as YY1 acting as a component responsible for a binding of the complex to the gene promoter. The formation of a similar complex, responsible for the silencing of human CCAAT/enhancer-binding protein delta, has already been reported in human cancer cell lines [35]. Consequently, it suggests that the epileptogenesis-evoked demethylation of the *Mmp-9* proximal promoter and removal of H3K27me3 from its chromatin are strictly related to the dissociation of this multisubunit complex.

We have previously showed, in the model of the PTZ-evoked-enhanced neuronal depolarization, that YY1 is a critical repressor of Mmp-9 expression in hippocampus [8]. Here, our results suggest that upregulation of hippocampal Mmp-9 expression during epileptogenesis is also YY1-dependent; however, it occurs in a different context of the histone modifications. Whereas in depolarized hippocampus activation of Mmp-9 expression is accompanied by the hyperacetylation of histones H3 and H4 in the genome fragment, during epileptogenesis and in epileptic hippocampus this phenomenon is histone hyperacetylation–independent and related to the accumulation of H3K27me3 and H3S10ph.

We have demonstrated that demethylation of *Mmp-9* proximal promoter during epileptogenesis is Gadd45β-dependent. Gadd45β is expressed in the brain, mostly in neurons [36]. It regulates active DNA demethylation in the brain [24] and influences synaptic plasticity [37]. It is considered an immediate early gene in neurons [24], and is upregulated by different neuronal activity and neural plasticity events [24, 37–40]. Unlike, we found that during another neuronal plasticity related process–the PTZ-evoked epileptogenesis in hippocampus, Gadd45β was stably expressed, and despite this, it still exerted its neuronal plasticity-evoked demethylation activity on the Mmp-9 gene promoter regulating its expression, and as a consequence the Mmp-9 related neuronal plasticity.

We also found that the transcriptional modification H3S10ph is strongly and gradually accumulated during epileptogenesis in hippocampus. H3S10ph is mostly involved in transcriptional activation and chromatin decondensation; however, sometimes it can be related to gene repression as well [41–42]. In Mmp-9 proximal gene chromatin context during hippocampal epileptogenesis, H3S10ph clearly acts like a transcriptionally activating molecular mark. Moreover, H3S10ph, especially in non-dividing cells, is usually very transient and depends on the presence of appropriate modifying kinases, for instance PKA or RSK2 [41]. Surprisingly, we see its stable upregulation in our study model in hippocampus, which is probably a consequence of deregulated and constantly activated kinase systems in epileptic rodents. Some kinases which can be involved in this phosphorylation are shown to be aberrantly expressed during epileptogenesis [43–45], and inhibition of some of them can show antiseizure effects [46]. Interestingly, it has also been demonstrated that H3S10ph can inhibit DNA methylation in *Neurospora* [47]. It is in accordance with our results, which suggest that H3S10ph can be involved in DNA demethylation in mammals too.

The COMPASS/COMPASS-like complex exhibits the mono-, di- and trimethylating H3K4 methyltransferase activity [48]. Recently, Cheng *et al*. have demonstrated that the protein complex is involved in the MMP-9 transcriptional activation in lung cancer cells stimulating H3K4me2 and H3K4me3 accumulation in the chromatin of the MMP-9 promoter [49]. In contrast, our results have shown that the activating histone modification H3K4me2 is not present in the chromatin of the *Mmp-9* proximal promoter in the hippocampus of the unstimulated rats as well as partially and fully kindled animals (S6E Fig). It suggests that COMPASS/COMPASS-like complex is probably not involved in the MMP-9 activation during epileptogenesis.

None of the genome-wide studies found differential methylation of MMP-9 promoter or DNA methylation dependent regulation of MMP-9 expression both during epileptogenesis and in epilepsy in rodents and humans [50–52]. These reports suggested that most of the distinctly methylated CpG sites localized in the genomes of epileptic rodents and patients with temporal lobe epilepsy are hypermethylated [50–52]. Additionally, in epileptic rats, most of differential DNA methylation events were not associated with changes in gene expression [50]. Even more strikingly, in humans there was completely no association of DNA methylation changes found in temporal lobe epilepsy with alterations in protein coding gene expression [52]. In contrary, our results show that MMP-9 gene promoter is hypomethylated during epileptogenesis as well as in epileptic rat and human hippocampus, and even more interestingly, that changes in MMP-9 gene promoter methylation status are strictly related to alterations in its mRNA expression. Consequently, MMP-9 is the first and only gene for which this correlation is found in human temporal lobe epilepsy.

Kobow et al. [50] showed no changes in mRNA expression profiles of Dnmts by mRNA-Seq in the hippocampus of epileptic rats. Similarly, we found that the mRNA expression of DNA methyltransferases Dnmt1, Dnmt3a, and Dnmt3b does not change during PTZ-induced epileptogenesis in rat hippocampus. On the contrary, it has been shown that Dnmt1 and Dnmt3a expression is increased in the anterior temporal neocortex in human temporal lobe epilepsy [53]. This may indicate on interspecies or brain interregional differences in regulation of expression of DNA methyltransferases in epilepsy. Intriguingly, Kobow et al. [50] also showed that the H3K27 histone methyltransferase Ezh1 expression is decreased in epileptic rat hippocampus. It is in accordance with our observation of diminished accumulation of H3K27me3 in *Mmp-9* proximal promoter chromatin during epileptogenesis.

One should emphasize that in this study, we applied a new research approach to study epileptogenesis. We separated a unique study group of animals, which are not kindled, but are still during kindling (partially kindled study group). We believe that the group can give insight into early mechanisms of epilepsy development, which are very closely related to those of human epileptogenesis. The formation of a separate study group composed of partially kindled animals is critical, in our opinion, for the understanding of epileptogenesis and its dynamics. This approach to analyze the PTZ-kindling results is our original, completely innovative modification aiming to study mechanisms related to epileptogenesis. The approach allowed us to separate the molecular effects exerted by epileptogenesis from those related to epilepsy and epileptic activity. Consequently, it allows a disclosure of molecular mechanisms underlying causes, and not consequences, of epilepsy. Surprisingly, we did not find any reports in the literature presenting the approach. In the papers describing experiments performed on kindling models, authors compared only the control group of animals to the fully kindled one, and then drew conclusions from such experimental design, which we think is not sufficiently informative to study phenomena related to epileptogenesis. In our opinion, to infiltrate and illuminate the mechanisms of epilepsy development, one should concentrate on animals during kindling, rather than those which are already kindled, thus in other words which are epileptic.

Thanks to this separation of study groups in our PTZ-kindling model, we have showed that YY1 dissociation is an early molecular event during epileptogenesis-evoked Mmp-9 upregulation. Moreover, what is even more important is we have revealed that the molecular processes responsible for the upregulation of Mmp-9 expression are accumulated step-by-step during epileptogenesis. It suggests that they are rather phenomena related to epileptogenesis itself and not a consequence of epilepsy-induced pathology, which means that they are causes of epileptic phenotype and not a consequence of it. Similarly, there is still the unresolved question of whether changes in DNA methylation found in epilepsy are just a repercussion of the disease, and its seizure activity, or are one of the causes of this disorder. Our results challenge this question, suggesting that the dysregulation of DNA methylation found in epilepsy is a source, and not a consequence, of this condition.

## Supporting Information

S1 Fig Mmp-9 mRNA expression and promoter methylation in hippocampi isolated immediately and 72-hours after rat sacrifice. **(A)** For each analysis equal amounts of RNA samples isolated from the rat hippocampi immediately or 72-hours after rat sacrifice were used. Data are presented as fold change in mRNA expression. Values are means ± SEM (*n* = 4). The methylation level of the *Mmp-9* proximal promoter **(B)** was revealed using qPCR analyzing DNA samples obtained by MeDIP from rat hippocampi removed immediately or 72-hours after rat sacrifice. Data are presented as a fold change in *Mmp-9* proximal promoter methylation level. Values are means ± SEM (*n* = 4).
(TIF)

S2 Fig Hippocampal Mmp-9 mRNA expression and promoter methylation are not influenced by aging occurring during kindling. Rats began the PTZ-evoked kindling in age of 12 weeks and ended when were around 17 week old. **(A)** For each analysis equal amounts of RNA samples isolated from 12 week or 17 week old rat hippocampi were used. Data are presented as fold change in mRNA expression. Values are means ± SEM (*n* = 4). **(B)** The methylation level of the *Mmp-9* proximal promoter was revealed using qPCR analyzing DNA samples obtained by MeDIP from 12 week or 17 week old rat hippocampi. Data are presented as a fold change in *Mmp-9* proximal promoter methylation level. Values are means ± SEM (*n* = 4).
(TIF)

S3 Fig Dnmt1, Dnmt3a and Dnmt3b are stable expressed during epileptogenesis in the rat hippocampus. 30 mg/kg of PTZ was administrated intraperitoneally at least 10 times to partially kindled and fully kindled study group. Rats were sacrificed 24 h after the final dose. For each analysis equal amounts of RNA samples isolated from naive (control) and PTZ-treated (partially kindled, full kindled) rat hippocampi were used. Data are presented as fold change in mRNA expression. Values are means ± SEM (*n* = 4).
(TIF)

S4 Fig Gadd45α and Gadd45β are stable expressed during epileptogenesis in the rat hippocampus. 30 mg/kg of PTZ was administrated intraperitoneally at least 10 times to partially kindled and fully kindled study group. Rats were sacrificed 24 h after the final dose. For each analysis equal amounts of RNA samples isolated from naive (control) and PTZ-treated (partially kindled, fully kindled) rat hippocampi were used. Data are presented as fold change in mRNA expression. Values are means ± SEM (*n* = 4).
(TIF)

S5 Fig H3K9me2 and H3K9me3 are not significantly enriched in chromatin of *Mmp-9* proximal promoter during epileptogenesis. DNA was isolated from hippocampal samples obtained by chromatin immunoprecipitation with anti-H3K9me2 or anti-H3K9me3 antibodies from the unstimulated (control), as well as the partially kindled and fully kindled rats. The *Mmp-9* proximal promoter content was evaluated by qPCR. Control ChIP reaction was performed using isotype antibody. Values are means ± SEM (*n* = 4).
(TIF)

S6 Fig Hyperacetylated histone H3 or H4, H3K9ac and H3K4me2 in *Mmp-9* chromatin during epileptogenesis. (**A) *During epileptogenesis in the rat hippocampus*, *the histone H3 is hypoacetylated*, *whereas the histone H4 is stably hyperacetylated*.** Equal amounts (20μg) of nuclear cell lysates obtained from the unstimulated (control) as well as the partially and fully kindled rat hippocampi were analyzed by Western blot with anti-H3ac and anti-H4ac antibodies. As a loading control, histone H3 was used. Representative Western blot analyses are shown. For Suppl. Fig 6B–6D DNA was isolated from hippocampal samples obtained by chromatin immunoprecipitation from the unstimulated (control), as well as the partially and fully kindled rats, with the following antibodies: anti-H3ac for Suppl. Fig 6B, anti-H4ac for Suppl. Fig 6C, anti-H3K9ac for Suppl. Fig 6D, anti-H3K4me2 for Suppl. Fig 6E. *Mmp-9* proximal promoter content was evaluated by qPCR. Control ChIP reaction was performed using isotype antibody. For Suppl. Fig 6B–6D values are means ± SEM (*n* = 4). (**B) *The histone H3 is not hyperacatylated in the chromatin of Mmp-9 proximal promoter during epileptogenesis in the rat hippocampus in vivo*. (C) *The histone H4 is not hyperacatylated in the chromatin of Mmp-9 proximal promoter during epileptogenesis in the rat hippocampus in vivo*. D, *H3K9ac is not present in the chromatin of Mmp-9 proximal promoter during epileptogenesis in the rat hippocampus in vivo*. E, *H3K4me2 is not present in the chromatin of Mmp-9 proximal promoter during epileptogenesis in the rat hippocampus in vivo*.**
(TIF)

S1 Table Antibodies used for WB and ChIP. (DOCX)

S2 Table Primer sequences used for RT-qPCR and ChIP as well as applied PCR conditions. (DOCX)

S3 Table Primer sequences used for MeDIP, hMeDIP, BC, BSP, MSP and applied PCR conditions. (DOCX)
