# Supplementary figures and images for: Epigenetics of Epileptogenesis-Evoked Upregulation of Matrix Metalloproteinase-9 in Hippocampus
Source: PLoS One. 2016 Aug 9;11(8):e0159745. doi: 10.1371/journal.pone.0159745 (PMC4978505; doi:10.1371/journal.pone.0159745)

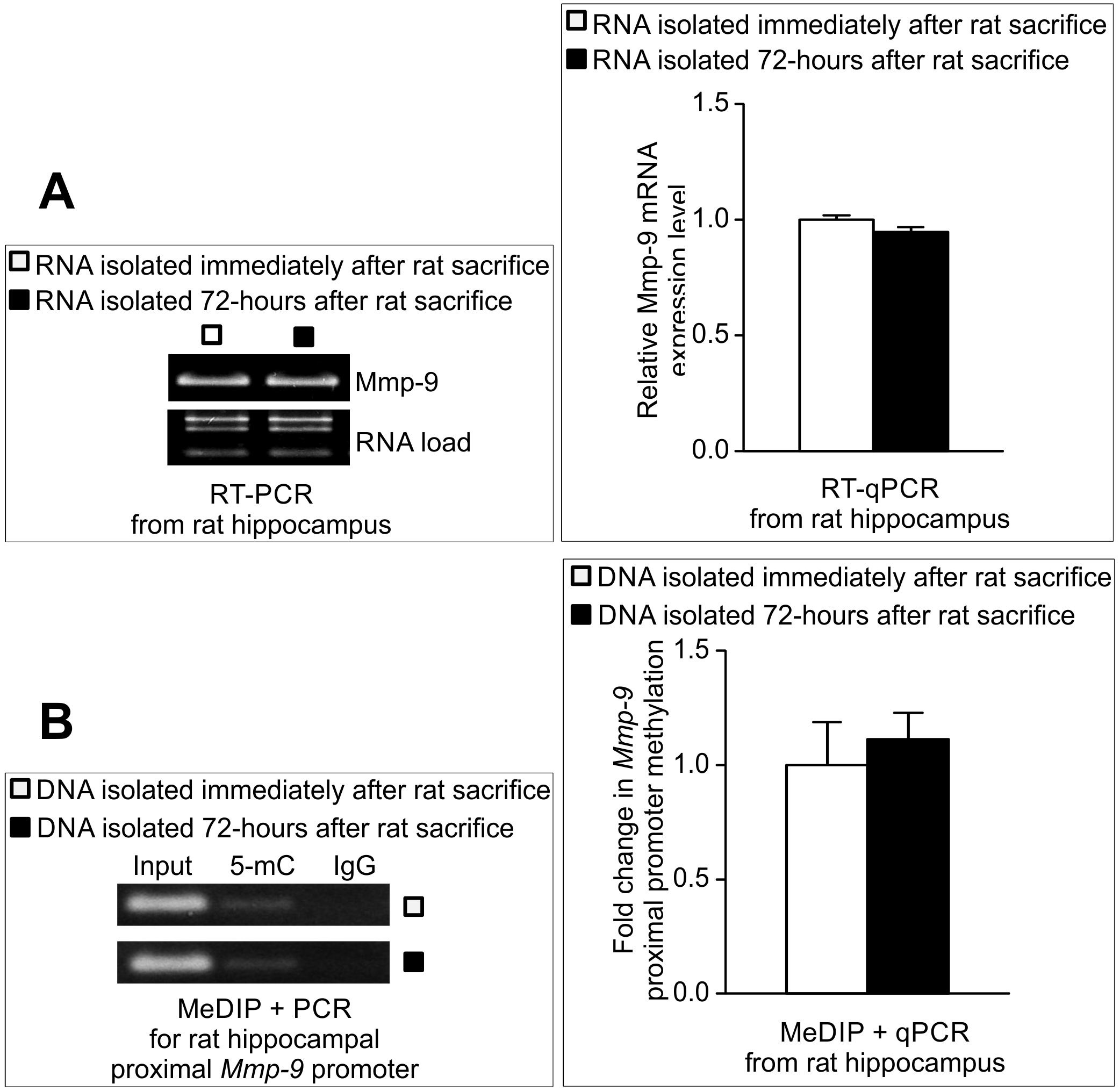

Supplement: S1 Fig — (A) For each analysis equal amounts of RNA samples isolated from the rat hippocampi immediately or 72-hours after rat sacrifice were used. Data are presented as fold change in mRNA expression. Values are means ± SEM (n = 4). The methylation level of the Mmp-9 proximal promoter (B) was revealed using qPCR analyzing DNA samples obtained by MeDIP from rat hippocampi removed immediately or 72-hours after rat sacrifice. Data are presented as a fold change in Mmp-9 proximal promoter methylation level. Values are means ± SEM (n = 4). (TIF) [file pone.0159745.s001.tif]

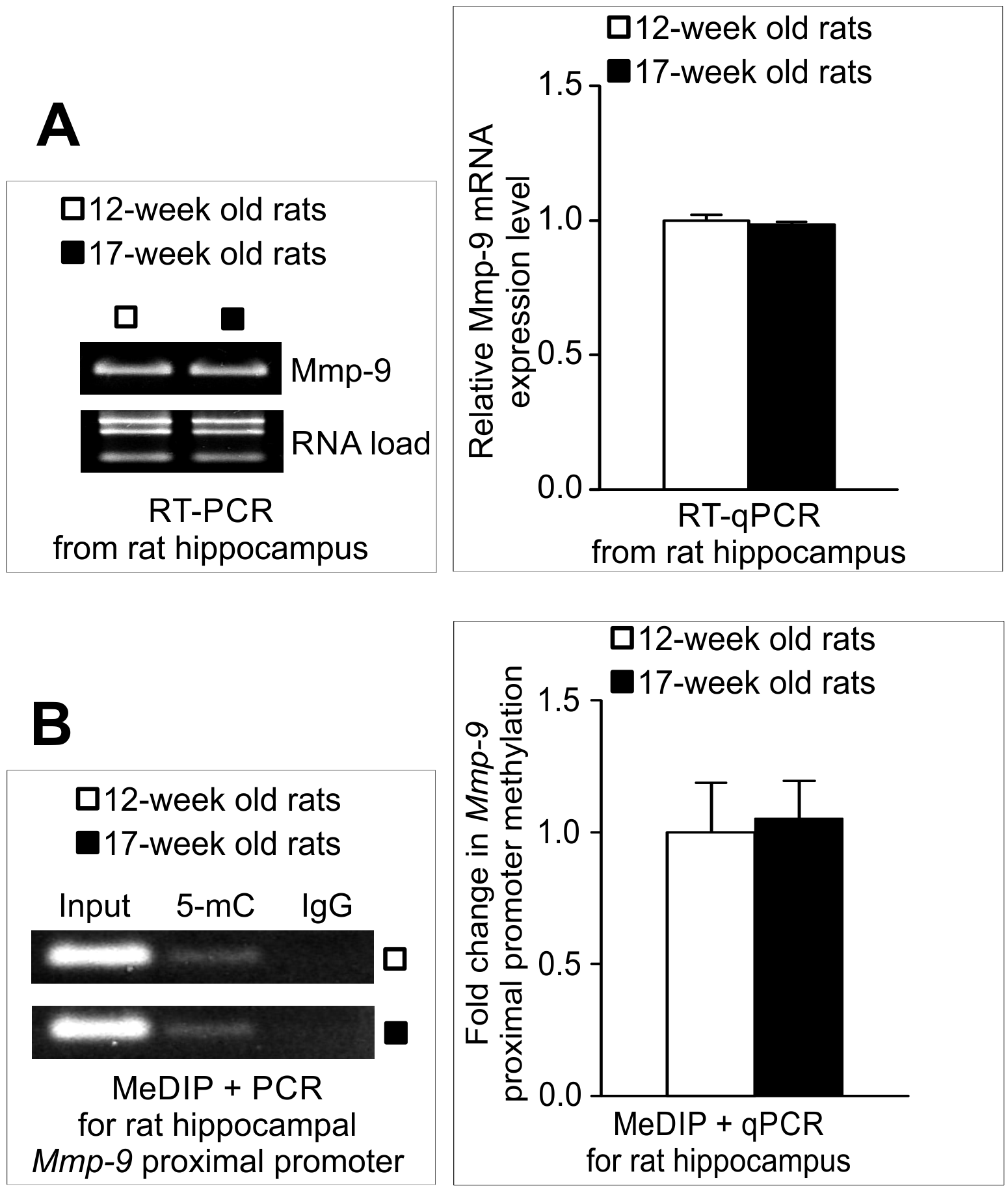

Supplement: S2 Fig — Rats began the PTZ-evoked kindling in age of 12 weeks and ended when were around 17 week old. (A) For each analysis equal amounts of RNA samples isolated from 12 week or 17 week old rat hippocampi were used. Data are presented as fold change in mRNA expression. Values are means ± SEM (n = 4). (B) The methylation level of the Mmp-9 proximal promoter was revealed using qPCR analyzing DNA samples obtained by MeDIP from 12 week or 17 week old rat hippocampi. Data are presented as a fold change in Mmp-9 proximal promoter methylation level. Values are means ± SEM (n = 4). (TIF) [file pone.0159745.s002.tif]

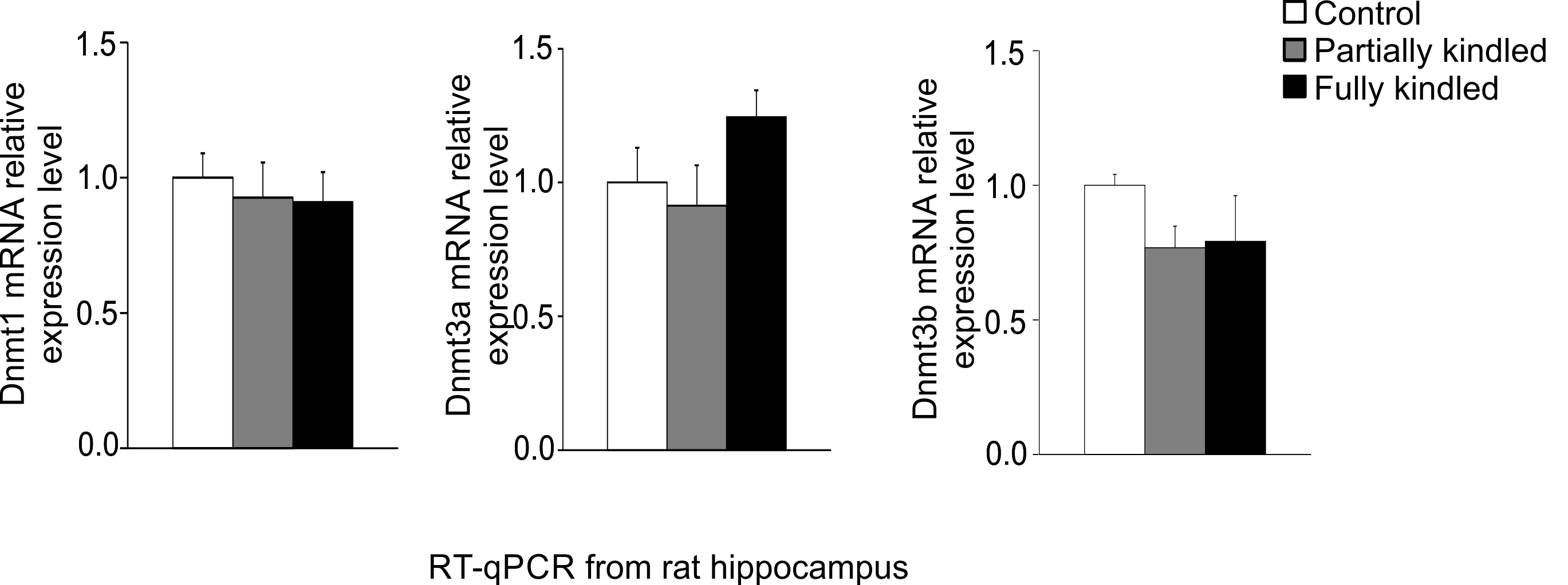

Supplement: S3 Fig — 30 mg/kg of PTZ was administrated intraperitoneally at least 10 times to partially kindled and fully kindled study group. Rats were sacrificed 24 h after the final dose. For each analysis equal amounts of RNA samples isolated from naive (control) and PTZ-treated (partially kindled, full kindled) rat hippocampi were used. Data are presented as fold change in mRNA expression. Values are means ± SEM (n = 4). (TIF) [file pone.0159745.s003.tif]

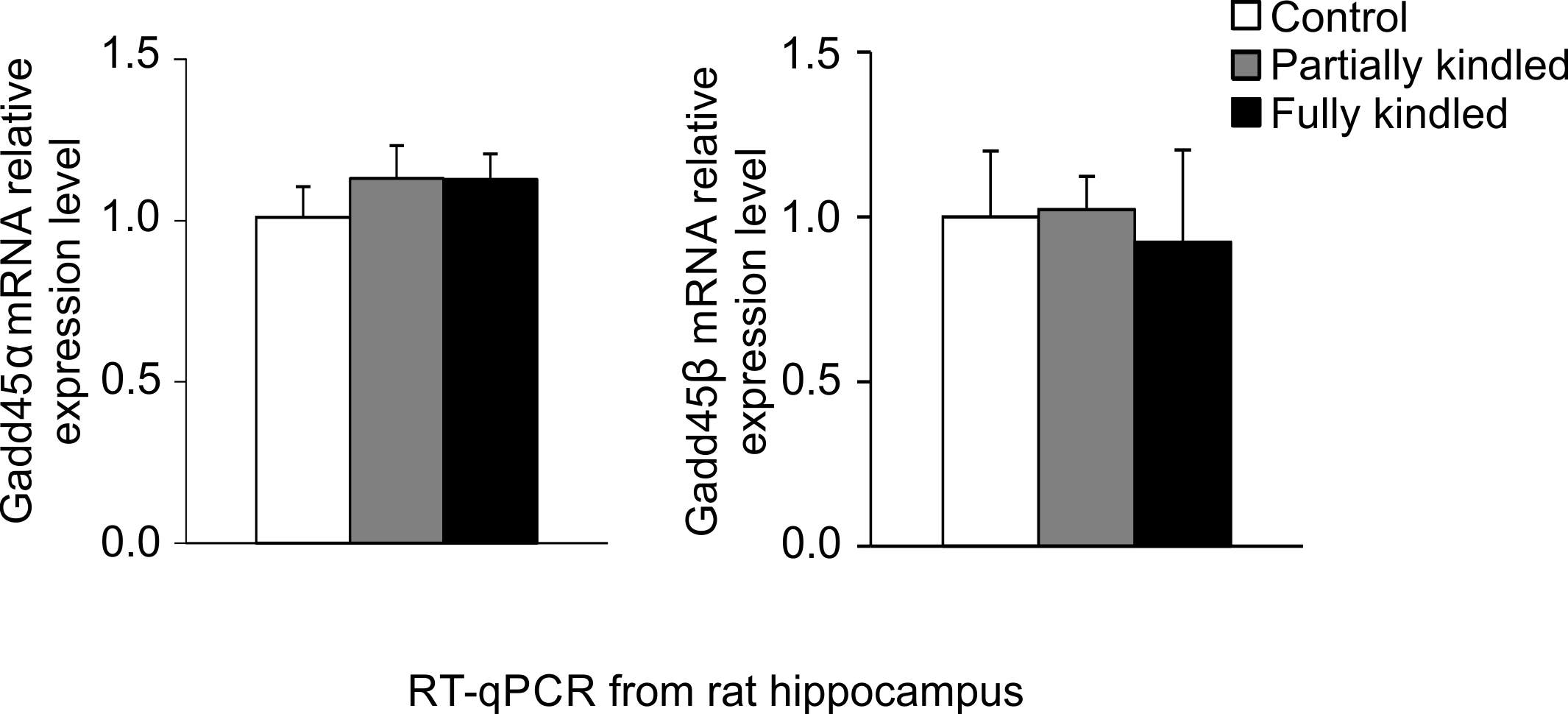

Supplement: S4 Fig — 30 mg/kg of PTZ was administrated intraperitoneally at least 10 times to partially kindled and fully kindled study group. Rats were sacrificed 24 h after the final dose. For each analysis equal amounts of RNA samples isolated from naive (control) and PTZ-treated (partially kindled, fully kindled) rat hippocampi were used. Data are presented as fold change in mRNA expression. Values are means ± SEM (n = 4). (TIF) [file pone.0159745.s004.tif]

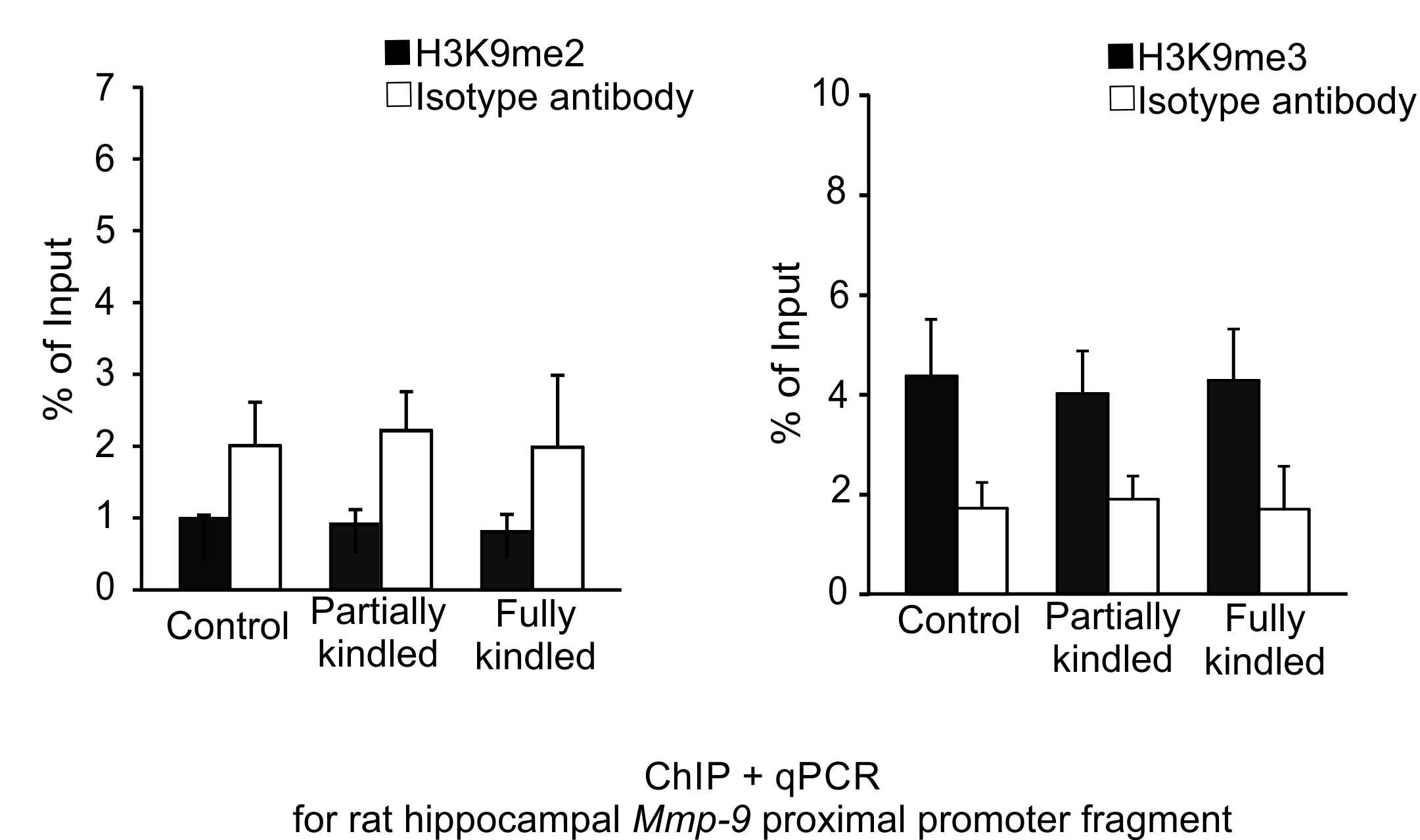

Supplement: S5 Fig — DNA was isolated from hippocampal samples obtained by chromatin immunoprecipitation with anti-H3K9me2 or anti-H3K9me3 antibodies from the unstimulated (control), as well as the partially kindled and fully kindled rats. The Mmp-9 proximal promoter content was evaluated by qPCR. Control ChIP reaction was performed using isotype antibody. Values are means ± SEM (n = 4). (TIF) [file pone.0159745.s005.tif]

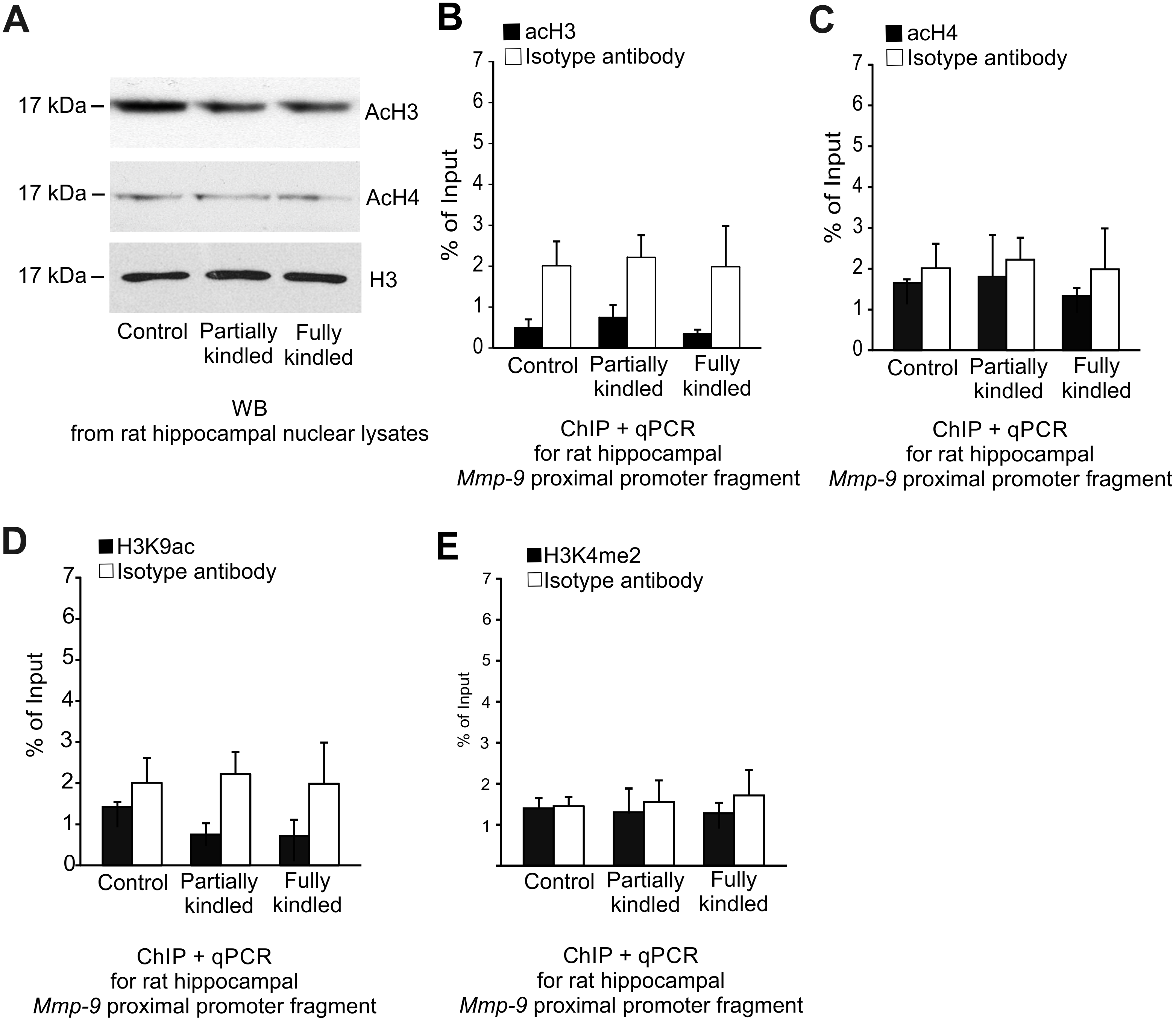

Supplement: S6 Fig — (A) During epileptogenesis in the rat hippocampus, the histone H3 is hypoacetylated, whereas the histone H4 is stably hyperacetylated. Equal amounts (20μg) of nuclear cell lysates obtained from the unstimulated (control) as well as the partially and fully kindled rat hippocampi were analyzed by Western blot with anti-H3ac and anti-H4ac antibodies. As a loading control, histone H3 was used. Representative Western blot analyses are shown. For Suppl. Fig 6B–6D DNA was isolated from hippocampal samples obtained by chromatin immunoprecipitation from the unstimulated (control), as well as the partially and fully kindled rats, with the following antibodies: anti-H3ac for Suppl. Fig 6B, anti-H4ac for Suppl. Fig 6C, anti-H3K9ac for Suppl. Fig 6D, anti-H3K4me2 for Suppl. Fig 6E. Mmp-9 proximal promoter content was evaluated by qPCR. Control ChIP reaction was performed using isotype antibody. For Suppl. Fig 6B–6D values are means ± SEM (n = 4). (B) The histone H3 is not hyperacatylated in the chromatin of Mmp-9 proximal promoter during epileptogenesis in the rat hippocampus in vivo. (C) The histone H4 is not hyperacatylated in the chromatin of Mmp-9 proximal promoter during epileptogenesis in the rat hippocampus in vivo. D, H3K9ac is not present in the chromatin of Mmp-9 proximal promoter during epileptogenesis in the rat hippocampus in vivo. E, H3K4me2 is not present in the chromatin of Mmp-9 proximal promoter during epileptogenesis in the rat hippocampus in vivo. (TIF) [file pone.0159745.s006.tif]
