# Supplementary material for: Epigenetics of Epileptogenesis-Evoked Upregulation of Matrix Metalloproteinase-9 in Hippocampus
Source: PLoS One. 2016 Aug 9;11(8):e0159745. doi: 10.1371/journal.pone.0159745 (PMC4978505; doi:10.1371/journal.pone.0159745)
Supplement: S1 Table — (DOCX) [file pone.0159745.s007.docx]

| **Gene** | **Primers** | **Product length** | **PCR conditions** |
| --- | --- | --- | --- |
| **Methylated and Hydroxymethylated DNA Immunoprecipitation (MeDIP/hMeDIP)** **with subsequent qPCR** | | | |
| *MMP-9* proximal promoter (human) | F: 5’-CACCATCCGTTGCGGACTTAC-3’ | 175 bp | 45 cycles of 95°c for 10 sec, 60°C for 15 sec, and 72°C for 15 sec |
|  | R: 5’-CCTCGGGCAAATGTCTTACCAC-3’ |  |  |
| *Mmp-9* proximal promoter (rat) | F: 5’-CTTTGGGCTGCCCAACAC-3’ | 158 bp | 45 cycles of 95°c for 10 sec, 60°C for 15 sec, and 72°C for 15 sec |
|  | R: 5’-AGCAGAATTTGCGGAGGTTTT-3’ |  |  |
| **Bisulfite conversion (BC)** | | | |
| *Mmp-9* promoter (rat) - Unconverted DNA | F: 5’-GCTTACTGAAGGCACATTAAGACCC-3’ | 212 bp | 45 cycles of 95°c for 10 sec, 60°C for 15 sec, and 72°C for 15 sec |
|  | R: 5’-GGAAACTAGACTGGGGTCTTGGC-3’ |  |  |
| *Mmp-9* promoter (rat) - Converted DNA | F: 5’-GTTTATTGAAGGTATATTAAGATTT-3’ | 212 bp | 45 cycles of 95°c for 10 sec, 60°C for 15 sec, and 72°C for 15 sec |
|  | R: 5’-AAAAACTAAACTAAAATCTTAAC-3’ |  |  |
| **Subcloning of bisulfite converted DNA and sequencing (BS)** | | | |
| -272/+291bp fragment of rat Mmp-9 gene | F: 5’-TATTTTTTTGAGTGTTGTGGTTTTTT-3’ | 548 bp | 95°c for 4 min , 30 cycles of 95°c for 30 sec, 58°C for 30 sec, and 72°C for 40 sec, and 72ºC for 7 min |
|  | R: 5’-ACCTACCCCTACTACCTAACCCTAAC-3’ |  |  |
| -1243/-695 bp fragment of rat  *Mmp-9* promoter | F: 5’-TGGTGGATTTAGGATTGTAATTTAGT-3’ | 563 bp | 95°c for 4 min , 30 cycles of 95°c for 30 sec, 55°C for 30 sec, and 72°C for 40 sec, and 72ºC for 7 min |
|  | R: 5’-AAACCTTTATCCCTCAAACCTTTAT-3’ |  |  |
| **Methylation Specific PCR (MSP)** | | | |
| -98/-97 bp CpG site in *Mmp-9* (rat) promoter | F: 5’-GGGGATTGTGGGTAGGGTATAA-3’ | 131 bp | 95°C for 10 min, 30 cycles of 94°C for 15 sec, 59°C for 30 sec, and 72°C for 30 sec; followed by 72°C for 10 min |
|  | R_UM_: 5’-CCCTCCCTCCAAACTTACACTA-3’ |  |  |
|  | R_M_: 5’-CCCTCCCTCCAAACTTACGCTA-3’ |  |  |
| -319/-318 bp CpG site in *Mmp-9* (rat) promoter | F_UM_: 5’-GATGGTTTTTTTATTATGTGTA-3’ | 103 bp | 95°C for 10 min, 30 cycles of 94°C for 15 sec, 50°C for 30 sec, and 72°C for 30 sec; followed by 72°C for 10 min |
|  | F_M_: 5’-GATGGTTTTTTTATTATGCGTA-3’ |  |  |
|  | R: 5’-AAAAAACCACAACACTCAAAAA-3’ |  |  |
| -582/-581 and -680/  -679 bp CpG sites in *Mmp-9* (rat) promoter | F_UM_: 5’-TTTTTAAATAGAAGAGGAATGAT-3’ | 136 bp | 95°C for 10 min, 30 cycles of 94°C for 15 sec, 50°C for 30 sec, and 72°C for 30 sec; followed by 72°C for 10 min |
|  | F_M_: 5’-TTTTTAAATAGAAGAGGAACGAT-3’ |  |  |
|  | R_UM_: 5’-AATTTAAAAAAATTCCACAAAAC-3’ |  |  |
|  | R_M_: 5’-AATTTAAAAAAATTCCACGAAAC-3’ |  |  |
| β-tubulin-4 (rat):  no CpG-sites in primers sequences | F: 5’-GGAGAGTAATATGAATGATTTGGTG-3’ | 128 bp | 95°C for 10 min, 30 cycles of 94°C for 15 sec, 55°C for 30 sec, and 72°C for 30 sec; followed by 72°C for 10 min |
|  | R: 5’-CATCTCAACTTTCCCTAACCTACTTAA-3’ |  |  |

F - forward, R – reverse, UM – unmethylated DNA, M – methylated DNA
