# Supplementary material for: Epigenetics of Epileptogenesis-Evoked Upregulation of Matrix Metalloproteinase-9 in Hippocampus
Source: PLoS One. 2016 Aug 9;11(8):e0159745. doi: 10.1371/journal.pone.0159745 (PMC4978505; doi:10.1371/journal.pone.0159745)
Supplement: S2 Table — (DOCX) [file pone.0159745.s008.docx]

| **Gene** | **Primers** | **Product length** | **PCR conditions** |
| --- | --- | --- | --- |
| **Reverse Transcription Quantitative PCR (RT-qPCR)** | | | |
| MMP-9 (human) | F: 5’-CAGTCCACCCTTGTGCTCTTC-3’ | 278 bp | 45 cycles of 95°c for 10 sec, 62°C for 15 sec,  and 72°C for 15 sec |
|  | R: 5’-TGCCACTTGAGGTCGCCCTCA-3’ |  |  |
| GAPDH (human) | F: 5’-GAAGGTGAAGGTCGGAGTC-3’ | 226 bp | 45 cycles of 95°c for 10 sec, 62°C for 15 sec,  and 72°C for 15 sec |
|  | R: 5’-GAAGATGGTGATGGGATTTC-3’ |  |  |
| Mmp-9 (rat) | F: 5’-AAATGTGGGTGTACACAGGC-3’ | 309 bp | 45 cycles of 95°c for 10 sec, 55°C for 15 sec,  and 72°C for 15 sec |
|  | R: 5’-TTCACCCGGTTGTGGAAACT-3’ |  |  |
| β-actin (rat) | F: 5’-TCCTTCCTGGGTATGGAATC-3’ | 300 bp | 45 cycles of 95°c for 10 sec, 60°C for 15 sec,  and 72°C for 15 sec |
|  | R: 5’-ACTCATCGTACTCCTGCTTG-3’ |  |  |
| **Chromatin Immunoprecipitation (ChIP) with subsequent qPCR** | | | |
| *Mmp-9* proximal promoter (rat) | F: 5’-CTTTGGGCTGCCCAACAC-3’ | 158 bp | 45 cycles of 95°c for 10 sec, 60°C for 15 sec, and 72°C for 15 sec |
|  | R: 5’-AGCAGAATTTGCGGAGGTTTT-3’ |  |  |

F - forward, R – reverse
