# Supplementary material for: Epigenetics of Epileptogenesis-Evoked Upregulation of Matrix Metalloproteinase-9 in Hippocampus
Source: PLoS One. 2016 Aug 9;11(8):e0159745. doi: 10.1371/journal.pone.0159745 (PMC4978505; doi:10.1371/journal.pone.0159745)
Supplement: S3 Table — (DOCX) [file pone.0159745.s009.docx]

| **Antibody** | **Supplier, catalog number** | **Method (dilution used) for WB)** |
| --- | --- | --- |
| H2Ak119ub1 | Upstate (Millipore), 05-678 | ChIP |
| H3 | Upstate (Millipore), 06-755 | WB (1:1000), ChIP |
| H3K4me2 | Upstate (Millipore), 05-1338 | ChIP |
| H3K9me2 | Upstate (Millipore), 07-441 | ChIP |
| H3K9me3 | Upstate (Millipore), 07-442 | ChIP |
| H3K27me3 | Upstate (Millipore), 07-449 | WB (1:1000), ChIP |
| H3S10ph | Upstate (Millipore), 06-570 | WB (1:1000), ChIP |
| H3ac | Upstate (Millipore), 06-599 | WB (1:1000), ChIP |
| H3K9ac | Upstate (Millipore), 07-352 | ChIP |
| H4ac | Upstate (Millipore), 06-866 | WB (1:1000), ChIP |
| Gadd45α (C-20) | Santa Cruz Biotechnology, sc-792 | WB (1:200), ChIP |
| Gadd45β (C-18) | Santa Cruz Biotechnology, sc-8776 | WB (1:200), ChIP |
| Dnmt1 (H-300) | Santa Cruz Biotechnology, sc-20701 | WB (1:500), ChIP |
| Dnmt3a (H-295) | Santa Cruz Biotechnology, sc-20703 | WB (1:500), ChIP |
| Dnmt3b (H-230) | Santa Cruz Biotechnology, sc-20704 | WB (1:500), ChIP |
| YY1 | Santa Cruz Biotechnology, sc-7341 | WB (1:200), ChIP |
| TBP | Pierce (Thermo Scientific), MA5-14739 | WB (1:1000) |
| normal mouse IgG | Santa Cruz Biotechnology, sc-2025 | ChIP |
| normal rabbit IgG | Santa Cruz Biotechnology, sc-2027 | ChIP |

WB – Western Blot; ChIP – Chromatin Immunoprecipitation
